# Towards *in vivo* g-ratio mapping using MRI: Unifying myelin and diffusion imaging

**DOI:** 10.1016/j.jneumeth.2020.108990

**Published:** 2021-01-15

**Authors:** Siawoosh Mohammadi, Martina F. Callaghan

**Affiliations:** a Department of Systems Neuroscience, University Medical Center Hamburg-Eppendorf, Hamburg, Germany; b Department of Neurophysics, Max Planck Institute for Human Cognitive and Brain Sciences, Leipzig, Germany; c Wellcome Centre for Human Neuroimaging, UCL Queen Square Institute of Neurology, University College London, UK

**Keywords:** g-ratio, Diffusion MRI, Magnetisation transfer imaging, Myelin water imaging, Multi-parameter mapping, *In-vivo* histology using MRI, Biophysical modelling, Myelin, axon, and fiber volume fractions

## Abstract

**Background:**

The g-ratio, quantifying the comparative thickness of the myelin sheath encasing an axon, is a geometrical invariant that has high functional relevance because of its importance in determining neuronal conduction velocity. Advances in MRI data acquisition and signal modelling have put *in vivo* mapping of the g-ratio, across the entire white matter, within our reach. This capacity would greatly increase our knowledge of the nervous system: how it functions, and how it is impacted by disease.

**New method:**

This is the second review on the topic of g-ratio mapping using MRI.

**Results:**

This review summarizes the most recent developments in the field, while also providing methodological background pertinent to aggregate g-ratio weighted mapping, and discussing pitfalls associated with these approaches.

**Comparison with existing methods:**

Using simulations based on recently published data, this review reveals caveats to the state-of-the-art calibration methods that have been used for *in vivo* g-ratio mapping. It highlights the need to estimate both the slope and offset of the relationship between these MRI-based markers and the true myelin volume fraction if we are really to achieve the goal of precise, high sensitivity g-ratio mapping *in vivo*. Other challenges discussed in this review further evidence the need for gold standard measurements of human brain tissue from *ex vivo* histology.

**Conclusions:**

We conclude that the quest to find the most appropriate MRI biomarkers to enable *in vivo* g-ratio mapping is ongoing, with the full potential of many novel techniques yet to be investigated.

## Highlights

• Second review on the topic of g-ratio mapping using MRI.
• A summary of the most recent developments in the fieldproviding methodological background.
• Discussion of pitfalls associated with g-ratio mapping using MRI.

## 1 Introduction

**Fig. 1:**
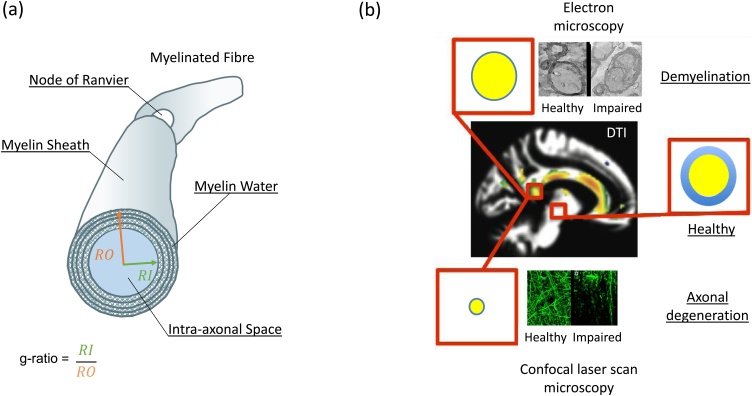
Illustration of how the MR g-ratio can facilitate non-invasive imaging of specific microscopic processes aka *in vivo histology using MRI*. (a) Schematic of a myelinated axon. (b) Coloured regions in a whole-brain DTI image highlighting where significant reduction of factional anisotropy (FA, adapted from (Teipel et al., 2015)) had been identified in patients with Alzheimer’s disease relative to healthy controls. While DTI is sensitive to neurodegenerative microstructural changes, it is not specific. This is illustrated by two well-known disease mechanisms that can lead to the same observed reduction in FA: (top) demyelination and (bottom) axonal degeneration. Today, these disease mechanisms can only be disentangled post-mortem using *ex vivo* histology, *e.g.*: electron microscopy for demyelination (top) or confocal laser scanning microscopy of axonal tracers for axonal degeneration (bottom). Note, these images were taken from human tissue for which there is no specific diagnosis of disease. The difference in tissue quality of the images are most probably caused by autolysis rather than pathology processes but were declared as “healthy” and “impaired” for illustration purposes only. The MR g-ratio (red box), together with its constituents: axonal volume fraction (AVF) in yellow and myelin volume fraction (MVF) in blue, can disentangle these two mechanisms noninvasively: while demyelination would only reduce MVF and thus increase the MR g-ratio, axonal degeneration would reduce both, MVF and AVF, and leave the MR g-ratio potentially unaffected.

The g-ratio is a geometrical invariant of axons quantifying their degree of myelination relative to their cross-sectional size. It is computed as the ratio of the inner axonal diameter, or radius, relative to that of the axon plus the myelin sheath that encases it (Fig. 1a). Coupled with the axonal diameter, the g-ratio is a key determinate of neuronal conduction velocity (Rushton, 1951; Chomiak and Hu, 2009; Schmidt and Knösche, 2019). Signal transmission along different axonal fibres can be regulated and synchronised by varying the degree of myelination, and therefore the g-ratio, to optimize cognitive function, sensory integration and motor skills (Fields, 2015). As the central nervous system appears to communicate at physical limits to constrain metabolic demands (Salami et al., 2003; Hartline and Colman, 2007; Coggan et al., 2015), small deviations from the optimal g-ratio value (0.6−0.8, (Rushton, 1951; Chomiak and Hu, 2009)) may have strong functional impact.

Until recently, information about axonal features, such as their g-ratio, have only been accessible by invasive methods such as *ex vivo* electron microscopy (Hildebrand and Hahn, 1978), which restricted analyses to small numbers of axons and a limited number of small brain regions or pathways. The g-ratio measured by such techniques is denoted the microscopic g-ratio because of the extremely fine spatial resolution that can be achieved. Clearly, using MRI to investigate the g-ratio *in vivo* would be highly desirable as it could provide whole brain information on a voxel-wise basis. Stikov et al. proposed the methodology by which such a non-invasive MR-based “aggregate” g-ratio could be measured (Stikov et al., 2011, 2015), which we denote in this review interchangeably the “MR g-ratio” or “g-ratio mapping”. The MR g-ratio framework measures the ensemble average across a voxel of an underlying, unresolved, microstructural distribution of g-ratios. Making a strong assumption that the g-ratio is constant within a voxel, Stikov et al. demonstrated, *via* a geometrical plausibility argument (Stikov et al., 2011, 2015), that this aggregate MR g-ratio can be computed on a voxel-wise basis from the ratio of the myelin and axonal volume fractions (MVF and AVF respectively). Establishing this relation was important because both the MVF and AVF can be estimated by combining biophysical models (Alexander et al., 2019; Novikov et al., 2019) and quantitative MRI within a framework known as *in vivo* histology using MRI (Weiskopf et al., 2015). As compared to standard quantitative MRI techniques, such as diffusion tensor imaging (DTI), the MR g-ratio and its constituents, available *via in vivo* histology, are more specific to the tissue microstructure and thus make promising clinical biomarkers. For example, in Alzheimer’s disease (Teipel et al., 2015), the g-ratio is expected to increase if the underlying disease mechanism is solely driven by demeylination processes that only affect the myelin sheath (blue disk in Fig. 1b, top relative to the “healthy” case) but leave the axonal body intact (yellow circle, Fig. 1b, top). Whereas an axonal degeneration process can potentially leave the g-ratio unchanged, but affect both its constituents, *i.e*. the myelin sheath and axonal body (as illustrated by a smaller volume fraction of blue and yellow compartments in Fig. 1b, bottom). To differentiate such processes and understand their functional implications, clinical research and diagnostics would benefit greatly from the capacity to measure the g-ratio of fibre pathways *in vivo*.

The challenge for, and validity of, *in vivo* g-ratio mapping centres on how precisely and accurately the AVF and MVF can be measured with the chosen MRI techniques. Three years ago, Campbell et al. thoroughly reviewed the methods of g-ratio mapping and highlighted potential pitfalls (Campbell et al., 2018a). A key outcome of their review was the introduction of the qualifying term “weighted” into the name MR g-ratio, *i.e*. aggregated g-ratio ***weighted*** mapping. They proposed this qualifier to acknowledge the impact that any miscalibration between the MR-based myelin proxy and the true MVF would have. Typically, *ex vivo* electron microscopy (EM) measures of the MVF act as the gold standard for methodological assessment and calibration.

Despite the challenges associated with accurate measurement and calibration of the MVF and AVF, many studies have exploited the potential of *in vivo* g-ratio weighted imaging for a variety of different applications (see Table 1 for full details). These have ranged from g-ratio mapping in infants (Melbourne et al., 2016) and children (Dean et al., 2016) to healthy adults (Mohammadi et al., 2015; Mancini et al., 2018; Berman et al., 2019; Drakesmith et al., 2019a), during healthy aging (Cercignani et al., 2017; Berman et al., 2018) and as a result of pathological change (Hagiwara et al., 2017; Hori et al., 2018; Kamagata et al., 2019; Yu et al., 2019).

**Table 1:** Summary of *in vivo* MR g-ratio mapping studies. Limitations associated with the biomarkers for MVF (LM) and AVF/FVF (LA) are summarized in Table A2 (in Appendix B).

	Biomarkers		Subjects or Participants	Remarks
	Axonal or Fibre volume fraction (AVF or FVF)	Myelin volume fraction (MVF)		LA.x and LM.x refer to limitations pertinent, respectively, to the AVF or MVF measure used.
Stikov et al., 2011	DWI1 (DTI)	SPGR (qMT)	5C	First model relating g-ratio to MVF and AVF. It assumed constant g-ratio in a voxel, and parallel axons. Fractional anisotropy was related to FVF assuming parallel fibres. LA.1, LM.1, LM.9
Stikov et al., 2015	DWI2.5 (NODDI)	SPGR (qMT)	1C; 1 P; 1Mc;	Revised g-ratio model. In this model, the g-ratio is still assumed to be constant in a voxel but the model was extended to nonparallel axons. LA.3,LA.4,LM.1, LM.9
Mohammadi et al., 2015	DWI1 (TFD)	MPM with multi-echo SPGR (MTsat)	36C	First group study on g-ratio mapping using the MPM and DTI protocol as biomarkers for MVF and FVF. LA.2, LM.1, LM.2, LM.9
West et al., 2016	–	–	6M	Revised MR g-ratio model validated on volume fractions from electron microscopy, revealing that the MR g-ratio, constructed under the assumption of constant intra-voxel g-ratios, is in fact a fibre area-weighted average of the true distribution of microscopic g-ratios.
Melbourne et al., 2016	DWI2 (NODDI)	2D GRASE (MET2)	37PI	The g-ratio of preterm infants scanned at 27 and 58 weeks. LA.3, LA.4, LM.6, LM.7
Dean et al., 2016	DWI2 (NODDI)	SPGR & bSSFP (mcDESPOT)	18I	g-ratio index changes across childhood (3 months to 7.5 years of age).LA.3, LA.4, LM.3, LM.9
Hagiwara et al., 2017	DWI2 (NODDI)	SyMRI	20P	g-ratio index in patients with multiple sclerosis. MVF was estimated *via* the SyMRI model (Warntjes et al., 2016). LA.3, LA.4, LM.10
Duval et al., 2017	DWI20 (CHARMED)	SPGR (MTV)	9C	g-ratio index in human spinal cord. LA.6, LM.4, LM.1, LM.9
Cercignani et al., 2017	DWI2.4 (NODDI)	bSSFP (qMT)	38C	qMT was calculated *via* in-house software. B1+ correction was not reported. Change of g-ratio as a function of age. LA.3,LA.4, LM.1, LM.9
Ellerbrock and Mohammadi, 2018a	DWI1 (TFD), DWI2 (NODDI)	MPM with multi-echo SPGR (MTsat, MTV)	12C, 10C	Four different g-ratio index maps were compared in a scan-rescan experiment between two groups of subjects (12 and 10 subjects). LA.2, LA.3, LA.4, LM.1, LM.2, LM.9
Berman et al., 2018	DWI1 (DTI)	SPGR (MTV)	92C; M15*	Change of g-ratio as a function of age. LA.1, LM.1, LM.4, LM.9
Duval et al., 2018	As in (Stikov et al., 2011)	SPGR (MTV)	8C	Scan-rescan of g-ratio in spinal cord. LA.1, LM.1, LM.4, LM.9
Hori et al., 2018	DWI1 (NODDI)	MPM with multi-echo SPGR (MTsat)	24P	Clinical study: G-ratio maps of the spinal cord in Cervical Spondylotic Myelopathy. LA.3, LA.4, LM.2, LM.9
Jung et al., 2018	DWI2 (NODDI)	Multi-echo SPGR (MET2*)	5C; 15M*	Two calibration methods for estimating MVF from myelin-water fraction. LA.3, LA.4, LM.1, LM.6, LM.8
Mancini et al., 2018	DWI2.4 (1), DWI2.9 (2) (NODDI)	bSSFP (1), SPGR (2) (qMT)	16C,15C	Same as in Cercignani et al., 2017. Two datasets, dataset one acquired at 1.5 T (1) and dataset two (2) at 3 T, each on a different imaging site. G-ratio used to introduce axonal myelination in connectomics. B1+ correction was not reported for site (1). LA.3, LA.4, LM.1, LM.9
West et al., 2018a	DWI6 (NODDI, WMTI, mcSMT)	3D MSE (MET2)	15M	Electron microscopy and *ex vivo* MRI of mouse models with varying degrees of myelination using multi-shell diffusion MRI and a 3D spin echo sequence. LA.3, LA.4, LA.5, LM.1, LM.6, LM.8
Kamagata et al., 2019	DWI2 (NODDI)	MPM with multi-echo SPGR (MTsat)	14C;14P	The brain network topology was assessed using g-ratio as a marker for the connectivity strength, comparison between healthy controls and patients with multiple sclerosis. LA.3, LA.4, LM.1, LM.2, LM.9
Yu et al., 2019	DWI17.8 (3CM)	SPGR (MTV)	19C; 30P	g-ratio and axon diameter mapping in patients with multiple sclerosis and healthy controls. LA.6, LM.4, LM.9
Berman et al., 2019	DWI1 (DTI)	SPGR (MTV)	37C	Estimating conduction velocities in fibre pathways using g-ratio and tractography in 37 subjects (20 younger and 17 older humans). LM.1, LM.4,LM.9
Drakesmith et al., 2019a	DWI6 (CHARMED)	SPGR & SSFP (mcDESPOT)	21C	Estimating conduction velocities in the corpus callosum using g-ratio and axon diameters. LA.6, LM.3,LM.9
Thapaliya et al., 2018	–	*Complex SPGR*	*10C*	*This method uses only relaxometry data and has yet to be compared to other methods combining myelin and diffusion MRI.*

C = health human controls; I = infants; M = mice; Mc = macaque; P = human patients; PI = preterm infants; 3CM = ActiveAx-like model (Alexander et al., 2010); * The mice data from (West et al., 2018a) were used.

The number that comes after DWI refers to the highest b-shell (in $ms/\mu m^{2}$) that was used in the experiment. The study of Thapaliya et al. is in italics to highlight that it is the only study that does not rely on combining two different MRI contrasts.

The review by Campbell et al. (Campbell et al., 2018a), increased awareness around the importance of calibrating the MVF proxy. Since then, a series of validation studies have been conducted by the Does lab (Kelm et al., 2016; West et al., 2018a, 2018b) based on extensive histological data and *ex vivo* MRI. These studies probed a broad dynamic range of MVF and g-ratio enabling insights into the validity and sensitivity of MR-based g-ratio mapping and its relationship with various MVF proxies. A number of methodological studies have also been published on g-ratio weighted mapping in recent years, e.g. to assess its repeatability (Duval et al., 2018; Ellerbrock and Mohammadi, 2018a), and the reproducibility when the particular proxies used for the AVF and MVF are varied (Ellerbrock and Mohammadi, 2018a).

In this review, we explore these methodological advances and seek to unify the nomenclature describing the various myelin and diffusion models. To do this, we provide the background to MRI methodologies that have been used to quantify the MVF and AVF (or fibre volume fraction, FVF) *in vivo*, focusing specifically on the techniques that have been used to date in the context of g-ratio mapping. We use the aforementioned validation studies in simulation-based experiments to further understand the impact of currently used calibration methods on the accuracy of the estimated MR g-ratio using three common myelin markers: the bound pool fraction, the macromolecular tissue volume, and the myelin water fraction. We conclude with an outlook on emerging approaches and what we think will be required to make g-ratio mapping with MRI a viable clinical tool.

## 2 Methodology

**Fig. 2:**
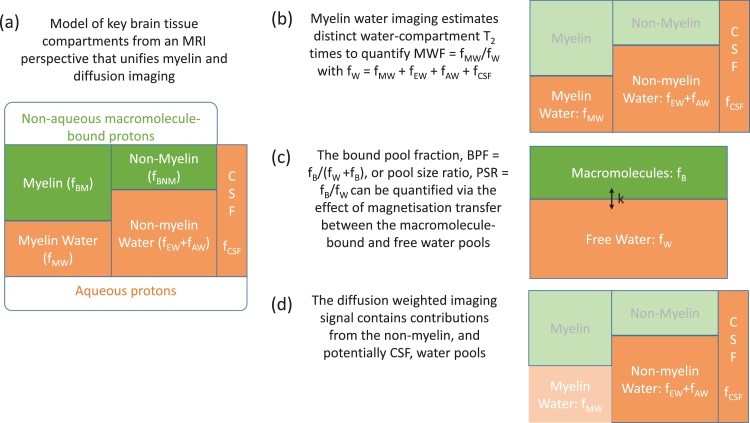
Unified nomenclature for myelin and axonal volume fraction imaging. To facilitate modelling, brain tissue is decomposed into four distinct tissue compartments (plus CSF) that are of key relevance from an MRI perspective. These cover two broad categories: non-aqueous macromolecule-bound (f_B_) and aqueous (f_W_) protons, each of which may (f_MW_, f_BM_) or may not (f_AW,_ f_EW,_ f_CSF,_ f_BNM_) be associated with myelin (a). Myelin water imaging specifically focuses on characterising the distinct water micro-environments, f_MW_, to quantify the myelin water fraction, MWF (b). Magnetisation transfer approaches focus instead on distinct macromolecular-bound and free water compartments, which can exchange magnetisation to quantify the bound pool fraction (BPF = f_B_/(f_B_+f_W_), c). The diffusion weighted signal is sensitive to intra-axonal and extra-axonal water compartments, and potentially to an isotropic diffusion compartment such as CSF. By decomposing the signal, the intra-axonal water fraction (AWF = f_AW_/(f_AW_+f_EW_+f_CSF_) can be isolated (d).

Biological tissue is formed of multiple microenvironments, which we refer to as compartments or pools. From an MRI perspective, key compartments in an imaging voxel comprised of human brain tissue, are those formed of aqueous and non-aqueous protons (Fig. 2a). The aqueous protons ($f_{\text{W}}$) appear in a variety of microenvironments including water trapped within the myelin sheaths of fibre pathways ($f_{\text{M}\text{W}}$), or contained within the intra- ($f_{\text{A}\text{W}}$) and extra-cellular spaces ($f_{\text{E}\text{W}}$), and cerebrospinal fluid ($f_{\text{C}\text{S}\text{F}}$). The non-aqueous protons are bound to macromolecules ($f_{\text{B}}$), including lipids and proteins in the myelin ($f_{\text{B}\text{M}}$) as well as in other macromolecules ($f_{\text{B}\text{N}\text{M}}$), e.g. glial cells. We express these compartments as fractions of the imaging voxel under the simplifying assumption that, while the relative contribution will spatially vary, every voxel is fully described by its content of water and bound protons, *i.e*. $f_{\text{W}}$ + $f_{\text{B}}$ = 1. Of these tissue compartments, it is the axonal and myelin-associated compartments that are important in the context of *in vivo* g-ratio mapping (Section 2.1). With MRI we tailor our experiments to maximise our sensitivity to specific compartments with the aim of quantifying the MVF and AVF respectively. To date, g-ratio mapping studies have either used relaxometry (Fig. 2b) or magnetisation transfer (Fig. 2c) techniques to quantify the myelin compartment (section 2.2), while mostly diffusion imaging has been used to quantify the axonal compartment (Fig. 2d and Section 2.3). These different imaging techniques have each evolved specific nomenclature over the course of their development. In this review, we aim, wherever possible, to unify these disparate notations using the fractional contributions outlined above and illustrated in Fig. 2.

### 2.1 The aggregate g-ratio model

**Fig. 3:**
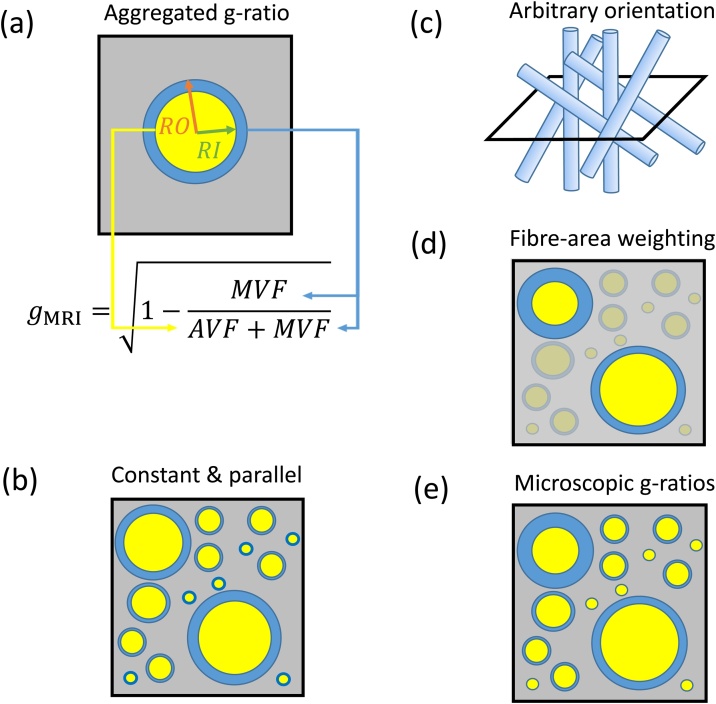
Schematic summary of the aggregated g-ratio model and its relation to the microscopic g-ratios. Myelinated axons are represented by cylindrical axonal (yellow) and annular myelin (blue) compartments (a–e), while other microstructural compartments are agglomerated in the background (grey). The aggregated g-ratio ($g_{MRI}$) can be formulated as a function of the axonal and myelin volume fractions (AVF and MVF respectively, a). In this model, all axons within a voxel are assumed to have the same g-ratio. In the initial model suggested by Stikov et al. in 2011, the axons were also assumed to be orientated in parallel (b). This assumption was subsequently relaxed (Stikov et al., 2015), allowing arbitrary axonal orientation (c). West et al. (2016) showed that the aggregated g-ratio is related to the fibre area-weighted mean of the microscopic g-ratios (e) – in the figure the weights are represented by the degree of transparency to indicate the weighting towards larger fibres (d).

Assuming a circular cross-section of axons, the microscopic g-ratio of an individual axon is defined as $g=\frac{RI}{RO}$, where RI and RO are the inner and outer radii of the fibre respectively (see Fig. 1a). All further considerations are targeting the white matter (WM), which is considered to be composed of three discrete, non-overlapping compartments: axonal, myelin, and extracellular space. In this case, any sample volume of WM can be described by the axonal (AVF), myelin (MVF), and extracellular (EVF) volume fractions of each compartment, which sum to one, *i.e*.: AVF+MVF+EVF=1. Using this WM model, Stikov and colleagues (Stikov et al., 2011, 2015) suggested that the aggregated g-ratio in an MRI volume (Fig. 3a) can also be defined in terms of volume fractions as:

(1) $$g_{\text{M}\text{R}\text{I}}=\sqrt{1-\frac{MVF}{MVF+AVF}}$$

To derive the relationship in Eq. (1) (see also (Stikov et al., 2011, 2015)), the g-ratio in an MRI voxel is assumed to be constant (Fig. 3b), whereas there is no restriction on the orientation of the axons in the voxel (Fig. 3c). Shortly after the g-ratio model was introduced, (West et al., 2016) suggested that $g_{MRI}$, which assumes constant intra-voxel g-ratio, is in fact capturing the fibre-area-weighted mean (Fig. 3d) of all the microscopic g-ratios within the voxel (Fig. 3e). If the assumptions of Eq. (1) hold, this model can also be used with other imaging modalities (e.g., electron microscopy, where the MVF and AVF have been measured after segmentation of the image (West et al., 2016). This efficient process allows the microscopic information obtained by these other modalities to be summarised over a spatial scale comparable to an MRI voxel, and therefore to be compared directly with the MR-based g-ratio in validation studies. The aggregate g-ratio model has been developed specifically for white matter (Stikov et al., 2011, 2015; Campbell et al., 2018a), where biomarkers of the MVF and AVF can be measured with MRI. In the following sections we will first outline the methods that have been used to date to quantify MVF and AVF in the context of g-ratio mapping.

### 2.2 Myelin volume fraction

A variety of different MRI-based measures have been used to characterise the myelin content within a voxel (Alonso‐Ortiz et al., 2015; MacKay and Laule, 2016; Sled, 2018). Here we focus on myelin-water imaging (MWI) and magnetization transfer (MT) imaging. In both cases, each of which will be discussed in turn, the measure aims to be reflective of the fractional myelin content within the imaging volume, *i.e*. the MVF. This is done by quantifying either the myelin *water* fraction ($MWF=\frac{f_{\text{M}\text{W}}}{f_{\text{W}}}$, Fig. 2b) or the bound pool fraction ($BPF=\frac{f_{\text{B}}}{f_{\text{W}}+f_{\text{B}}}$, Fig. 2c). In either case, an additional calibration step is clearly required to convert the measure to the MVF ($\frac{f_{\text{M}\text{W}}+f_{\text{B}\text{M}}}{f_{\text{W}}+f_{\text{B}}}$) in order to accurately compute the g-ratio (West et al., 2018b). As noted by Campbell et al. (Campbell et al., 2018a) this calibration step is crucial to the accuracy and precision of g-ratio mapping and will be discussed in detail in Section 3.

#### 2.2.1 MWF based on myelin water imaging

Starting from Fig. 2, the simplest water imaging model quantifies the density of free water protons within an imaging voxel, *i.e*. the proton density (PD) (Tofts, 2004). Under an assumption of complete longitudinal recovery within each repetition time, TR, the extrapolated MR signal at an echo time, TE, of 0 ms ($S_{0}$) is proportional to the product of the fractional water content, $f_{\text{W}}$, a calibration factor, C, that accounts for the concentration of protons in the voxel relative to that of free water, and the spatially-varying receive field sensitivity, R: $S_{0}=R\;C\;f_{\text{W}}$ such that $f_{\text{W}}+f_{\text{B}}=1$ (Fig. 2c). The receive field modulation must be estimated and removed ($S_{0}^{'}=C\;f_{\text{W}},$see Section 2.2.3) prior to final calibration, which is done with respect to a reference, e.g. cerebrospinal fluid, CSF: $PD=\frac{S_{0}^{'}}{S_{0,\text{C}\text{S}\text{F}}^{'}}=f_{\text{W}}$. This is equivalent to assuming that the volume fraction of macromolecules in CSF is zero (*i.e*. $f_{\text{B}}\approx 0$ and $f_{\text{W}}\approx 1$), *i.e*. $S_{0,\text{C}\text{S}\text{F}}^{'}=C$. The remaining contents of the voxel have recently been referred to as the macromolecular tissue volume ($MTV=1-PD=f_{\text{B}}$) (Mezer et al., 2013). Quantifying the fractional bound pool size in this manner assumes that the molar concentration of protons in macromolecules is the same as in free water, though it is estimated to be in the region of 15–20 % lower (West et al., 2018b). PD mapping typically makes no distinction between different water microenvironments (e.g. myelin water vs. non-myelin water) and instead estimates the sum of contributions from all compartments (Fig. 2b,c) under the assumption of a mono-exponential signal decay. Therefore, $f_{\text{B}}$ (or MTV) might vary with the minimum echo time, as well as the echo spacing, at which the signal was sampled (more details can be found in (Tofts, 2004)).

By contrast, myelin water imaging (MWI, (Alonso‐Ortiz et al., 2015)) extends this model to encompass multiple distinct water compartments, each with specific relaxation behaviour contingent on the local microenvironment. MWI quantifies myelin-associated aqueous protons in a voxel as a fraction of the total MR visible water signal, *i.e*. $MWF=\frac{f_{\text{M}\text{W}}}{f_{\text{W}}}$ as defined in Fig. 2b. To date, three main approaches to myelin water imaging have been used for g-ratio mapping using MRI (Table 1). Each technique exploits a different relaxation property to stratify the different tissue water compartments (MacKay and Laule, 2016): (1) multi-echo spin echo imaging to quantify compartment-specific transverse relaxation times (Melbourne et al., 2016; West et al., 2018a), T_2_, (2) multi-echo gradient echo imaging to quantify compartment-specific effective transverse relaxation times (Jung et al., 2018), T2*, and (3) multi-compartment driven equilibrium single pulse observation of T1 and T2 (mcDESPOT, (Deoni et al., 2008; Dean et al., 2016; Drakesmith et al., 2019a)) to distinguish fast and slow relaxing compartments based on their distinct T_1_ and T_2_ relaxation and exchange behaviour.

In MWI, the MWF is most commonly estimated by characterising the proportion of the water signal originating from different microstructural environments based on their distinct transverse relaxation times (T_2_). To do this, it is assumed that the residency time, τ, of the protons in each water pool is sufficiently long that their distinct relaxation behaviour can be discerned. The case τ >> T_2_ indicates a slow exchange regime, which can equivalently be described by an exchange rate k = 1/τ << 1/T_2_ (Zimmerman and Brittin, 1957). In this case, multi-exponential behaviour, with a component originating from each of the water pools having distinct amplitude and relaxation times, can be discerned. Indeed, T_2_ distributions from normal brain have been shown to contain multiple peaks that can be attributed to myelin water trapped between the lipid bilayers, intra/extracellular water and cerebral spinal fluid (Whittall et al., 1997; MacKay and Laule, 2016).

To quantify distinct T_2_ times, data are typically acquired using a multi-echo spin echo readout with a range of echo times. Each voxel is assumed to contain contributions from an unspecified number of slow or non-exchanging environments, each with distinct T_2_ decay times. Fitting the data to this model is typically done with a regularised non-negative least squares approach (Whittall and MacKay, 1989; MacKay et al., 2006), in which the regularisation ensures smoothly varying signal amplitudes as a function of T_2_. After fitting, the myelin compartment is assigned to the short T_2_ peaks, requiring a threshold T_2_ time to be specified. The MWF is then estimated as the area under the peaks below this threshold T_2_ time relative to the area under all peaks, *i.e*. $\frac{f_{\text{M}\text{W}}}{f_{\text{W}}}$ (MacKay et al., 2006). Clearly, the resulting pool size will vary depending on how this threshold, which will be field strength dependent, is set. MWI ignores any differential weighting that might be present, for example due to compartment-specific T_1_ times (Birkl et al., 2020). For software available for fitting such models, see e.g. (Doucette et al., 2020; markdoes, 2020).

In white matter, at least two different T_2_ relaxation times have been reported, which are associated with different tissue compartments (MacKay et al., 2006; Cercignani et al., 2018): (1) myelin water having a T_2_ of about 15–30 ms, and (2) water in the intra- and extra cellular spaces with a T_2_ of about 80–90 ms, at 3 T. It should also be noted that the T_2_ relaxation times of the intra- and extra cellular spaces likely differ (Dortch et al., 2013; Veraart et al., 2018; McKinnon and Jensen, 2019) and that there is exchange between these two compartments that also influences the T_2_ distribution in white matter (Sled et al., 2004). These effects will be revisited in Section 3.1.1 but have also been discussed in detail elsewhere (Does, 2018). A similar approach uses a multi-echo gradient echo acquisition in lieu of acquiring spin echoes. In this case compartment-specific T_2_* times are estimated instead of T_2_ (Lenz et al., 2012; Sati et al., 2013).

Rather than modelling distinct tissue compartments solely from the decay of the transverse magnetisation, the mcDESPOT approach integrates spoiled gradient echo (SPGR) and balanced steady-state free precision (bSSFP) images, acquired with different nominal flip angles, to fit a two compartment model of the steady state signal (Deoni et al., 2008). The combination of these two acquisition types allows both T_1_ (SPGR) and T_2_ (bSSFP) to be estimated (Deoni et al., 2013). In the mcDESPOT model distinct relaxation times are determined for a fast and a slow relaxing pool, as well as the exchange rate (k), or residency time (τ) of the two pools in the condition of thermal equilibrium (*i.e*. for two pools A and B, $k_{\text{A}\text{B}}M_{\text{A}}=k_{\text{B}\text{A}}M_{\text{B}}$, where $M_{\text{X}}$ is the magnetisation in the pool). The fast relaxing pool is subsequently assumed to be myelin-associated water allowing the MWF to be quantified. The relaxation and exchange of these two pools is modelled using the Bloch-McConnell equations, which allows analytical solutions for the steady state signal to be derived (McConnell, 1958; Liu et al., 2016). Fitting the acquired data to these signal models requires seven distinct model parameters to be estimated: T_1_, T_2_ and fractional amplitude for each compartment as well as the exchange between them.

#### 2.2.2 BPF based on magnetisation transfer

Like PD mapping, magnetisation transfer (MT) based approaches simplify the characterisation of white matter to two distinct pools (Fig. 2c). In this case one is comprised of an aqueous environment, $f_{\text{W}}$, and the other a non-aqueous environment, $f_{\text{B}}$, that, in the context of g-ratio mapping, is assumed to be associated with myelin. “Free” water, such as found within the intra- or extra-cellular compartments, has a sharp resonance linewidth, which is significantly broadened for the “bound” non-aqueous protons due to the restricted motion of this pool that leads to longer auto-correlation times and very short T_2_ in the range of tens of microseconds (Tofts, 2004). This means that the transverse magnetisation component is undetectable with MRI, unless ultra-short TE approaches are adopted (Sheth et al., 2016; Jang et al., 2020; Weiger et al., 2020), but also that the bound pool can be selectively saturated through the application of an off-resonance radiofrequency pulse prior to conventional excitation and signal detection. This pre-pulse can selectively saturate the longitudinal magnetisation of the bound pool while leaving the free pool largely unaffected. Subsequently, the process of magnetisation transfer (MT), primarily occurring through dipolar coupling between the bound and free pools, leads to an observable reduction in the measured signal intensity (Wolff and Balaban, 1989; Sled and Pike, 2001; Sled, 2018; van Zijl et al., 2018). MT techniques capture the proportion of magnetisation in the bound pool relative to the free pool through the pool size ratio ($PSR=\frac{f_{\text{B}}}{f_{\text{W}}}$ (Sled and Pike, 2001) and Fig. 2c) or, analogously to the MWF in MWI, relative to the total magnetisation in both pools *via* the bound pool fraction ($BPF=\frac{f_{\text{B}}}{f_{\text{W}}+f_{\text{B}}}$ (Sled, 2018) and Fig. 2c). In the first g-ratio mapping studies, the measured BPF was calibrated against histological data to convert it to an estimate of the MVF and combined with a diffusion-based measure of the FVF to estimate the g-ratio (Stikov et al., 2011, 2015).

The simplest means of probing the macromolecular bound pool *via* MT is to acquire an image using a pre-pulse with a single off-resonance frequency interleaved with a standard excitation pulse. The magnetisation transfer ratio (MTR) is defined as the normalised signal decrease relative to a reference image with only the standard excitation pulses (Henkelman et al., 2001). While this measure has been shown to be reflective of myelin content *via* histological analysis (Schmierer et al., 2004) it also depends on hardware, most notably the transmit field efficiency, B_1_^+^, and the T_1_ time, which reduces its comparability across individuals (Callaghan et al., 2015). Magnetisation transfer saturation (MTsat) incorporates corrections for both spatially varying T_1_ and B_1_^+^ effects to quantify the percent saturation per TR of the steady state SPGR signal that would result from a dual excitation sequence. This measure depends on the BPF (Helms et al., 2008), which has been verified empirically (Campbell et al., 2018a). It is also more robust to B_1_^+^ inhomogeneity than MTR (Callaghan et al., 2015). Note that, unlike MWF or BPF, MTsat is not a volume fraction and therefore always requires calibration (see Section 3.2 and Fig. 7b).

More comprehensive modelling of the two magnetisation pools is obtained through quantitative MT (qMT) imaging. This approach aims to separate the contributions of the free and bound pools by explicitly modelling the distinct T_1_ and T_2_ relaxation times of the pools and incorporating the exchange between them, under the assumption of thermal equilibrium. The absorption lineshape of the bound pool must also be modelled, and is often assumed to be super-Lorentzian, with a T_2_ in the region of tens of microseconds (Morrison and Henkelman, 1995). With this approach, the BPF can be estimated from the fractional magnetisation contributions of the two pools. To estimate this extended set of parameters, multiple images, sampling the so called z-spectrum, are acquired, each using a pre-pulse with a different off-resonance frequency (Sled and Pike, 2001; Cabana et al., 2015; Sled, 2018).

An intriguing, but not yet validated, approach that has also been used in the context of g-ratio mapping is to use multi-compartment Bloch simulations to model the myelin volume fraction within the voxel directly (Warntjes et al., 2016; Hagiwara et al., 2017).

#### 2.2.3 Protocol considerations for MVF mapping

Key protocol-specific limitations associated with the various approaches to quantifying the MVF that have been used to date in the context of g-ratio mapping are provided in Table A2 (in Appendix B).

Protocols to estimate the proton density, and by consequence the macromolecular tissue volume (Warntjes et al., 2007; Volz et al., 2012; Baudrexel et al., 2016; Mezer et al., 2016; Wang et al., 2018; Callaghan et al., 2019; Lorio et al., 2019) require an estimate of the receive field sensitivity, R, which can be obtained by constrained model fitting or measurement (Mezer et al., 2016). The normalisation step to express PD as a fraction, or more commonly a percentage, of the concentration of protons in pure water requires a reference region to be defined, e.g. within the CSF-filled ventricles. However, the optimal choice of the normalisation region will depend on the acquisition scheme since sufficient signal-to-noise rate (SNR) is required for robust estimation (CSF was used in (Berman et al., 2018) and white matter in (Ellerbrock and Mohammadi, 2018a)). The accuracy and precision of the PD estimation will in turn dictate the accuracy and precision of the MTV estimate. The mapping of PD was introduced in the context of fully relaxed signal (*i.e*. TR >> T_1_). However, for reasonable scan times, this requirement can be relaxed, but in this case it is necessary to correct for spatially varying T_1_ recovery. Any transverse decay must also be accounted for by extrapolating to a TE of 0 ms to prevent biases, e.g. under-estimation in regions with high iron content.

Multi-compartment MWI necessitates short echo times to adequately sample the decay of the short T_2_ myelin-associated water compartment but also sufficiently long echo times to capture slowly relaxing contributions such as CSF (Whittall et al., 1999; Wiggermann et al., 2020). This extends the minimum achievable TR and can lead to long acquisition times, particularly for spin echo based approaches, unless spatial coverage or resolution are sacrificed, though significant acceleration has recently been achieved using compressed sensing (Dvorak et al., 2020). Acquiring multiple spin echoes in a single readout increases temporal efficiency, but the train of pulses can lead to the refocusing of echoes from unwanted pathways, *i.e*. the production of stimulated echoes, when B_1_^+^ is inhomogeneous. Correction schemes based on simulating the impact of these echoes (e.g. (Lebel and Wilman, 2010)) have been proposed and can be incorporated into the fitting procedure. 2D slice-selective approaches are also vulnerable to magnetisation transfer and distorted slice profile effects. The latter can be mitigated either by modifying the sequence to ensure a sufficiently broad refocusing width, or by accounting for the effect during processing (Lebel and Wilman, 2010; Nöth et al., 2017). The large number of refocusing pulses also increases the specific absorption rate (SAR) of the sequence, which is particularly important with the move to ultra-high field (≥7 T). Gradient echo approaches quantifying T_2_* are generally more time efficient since characterising a shorter time constant, and are less demanding from a SAR perspective, but suffer from reduced SNR as a result of the more rapid decay. Complex-valued fitting can be particularly beneficial (Nam et al., 2015b) in addressing the general problem of Rician bias that results when fitting magnitude data with long echo times, where significant biases can be introduced and greatly alter the measured T_2_^(*)^ values (Bjarnason et al., 2013).

MTR and MTsat are time efficient means of quantifying the effect of magnetisation transfer. As highlighted earlier, MTsat is more hardware robust. In addition, high resolution maps can be obtained with whole brain coverage in reasonable scan times making it particularly appealing for clinical studies. This efficient method was used in the first group study mapping the g-ratio *in vivo* (Mohammadi et al., 2015). However, a limitation of these rapid approaches is that they are semi-quantitative. The saturation of the bound pool, and therefore of the free pool *via* magnetisation transfer, will depend on the particular off-resonance pulse used, most notably the power and offset frequency. For further details, acquisition protocols and software for estimating this parameter see e.g. (Tabelow et al., 2019).

qMT approaches circumvent this limitation by quantifying specific physical parameters. However, the extended datasets required to fit the full qMT model lead to a trade-off between scanning durations and spatial resolution and/or coverage. To constrain the model fits, parameters can be fixed, e.g. the T_1_ of the free and bound pools can be set equal to each other, or an “observed” T_1_ can be separately measured and integrated into the fitting to relate the T_1_ times of the bound and free pools. For further details and software available for fitting such models, see e.g. (Cabana et al., 2015).

Clearly, brain tissue can be characterised by a very broad range of physical parameters. The multi-parameter mapping (MPM) quantitative MRI protocol offers a comprehensive approach providing high resolution, whole brain estimates of (single compartment) T_1_, T_2_*, PD, MTV and MTsat, with correction for transmit and receive field effects, in clinically feasible scan times (Weiskopf et al., 2013; Callaghan et al., 2019; Tabelow et al., 2019). As such it provides simple proxies for both the macromolecular (*via* MTsat & MTV) and free water pools (PD) in a single protocol.

### 2.3 Axonal volume fraction and fibre volume fraction

Diffusion MRI is the method of choice to separate the intra- and extra-axonal tissue compartments ($f_{\text{A}\text{W}}$ and $f_{\text{E}\text{W}}$, Fig. 2d) because of the distinct diffusion properties of water in these compartments. However, as detailed above, the myelin-associated water compartment has a short T_2_. This means that diffusion-weighted MRI is insensitive to myelin water because of the comparatively long minimum echo time required to accommodate the application of diffusion gradients.

Although, there are several different diffusion-based approaches available to probe the intra-axonal tissue compartment (e.g. (Alexander et al., 2019; Novikov et al., 2019)), we will specifically focus on those approaches that have been used to date to estimate the intra-axonal volume fraction for the purpose of computing the aggregated g-ratio. These studies can be subdivided into two categories: the studies that have used standard DTI data and those that have used multi-shell (and even more advanced) diffusion MRI protocols. Each category will be discussed in turn.

#### 2.3.1 FVF from DTI data

The first category of g-ratio studies required only a limited set of measurement parameters, including only a single b-value and a modest number of diffusion directions, as defined by the DTI protocol because they refrained from explicitly modelling more than one tissue compartment. A feature of these studies was the interpretation of diffusion-MRI based measurements of the axonal compartment as the FVF rather than the AVF, which, given the insensitivity of the conventional diffusion MRI signal to the myelin water pool, is flawed as we will discuss further in the next section. Note that there is still an indirect contribution of myelin on the diffusion MRI signal, e.g., through the increase in diffusion anisotropy. This is why these models still show a correlation to FVF (Campbell et al., 2018b).

*DTI:* The first g-ratio mapping study by Stikov et al. (Stikov et al., 2011) used simulations, in which axons were modelled as straight, parallel cylinders to establish a second order relationship between the fractional anisotropy (FA) of the diffusion tensor and the total FVF. The assumption of straight and parallel cylinders, however, restricted the application of this model to white matter regions with well aligned fibres. As a result, it has only been applied to the corpus callosum to date (Stikov et al., 2011; Berman et al., 2018).

*TFD:* Again using a single b-value, the TFD was derived from fibre orientation distributions (Reisert et al., 2013) and assumed to be directly proportional to the FVF. This FVF model, which was first used by Mohammadi et al. (Mohammadi et al., 2015) for g-ratio mapping, is not restricted to well-aligned fibre pathways and thus could be applied across the whole brain. A proportionality constant that related TFD to FVF was combined with the calibration coefficient that related the MTsat myelin marker used to capture MVF and estimated by referencing against a ground truth g-ratio value from literature (Mohammadi et al., 2015). This calibration approach will be further discussed in the context of myelin biomarkers in Section 3.2. However, Ellerbrock et al. (Ellerbrock and Mohammadi, 2018a) recently showed the TFD-based FVF parameter to be less stable in terms of repeatability and comparability than FVF estimates derived from the Neurite and Orientation Dispersion in Diffusion Imaging (aka NODDI) model (Zhang et al., 2012), discussed in more detail in the next section.

#### 2.3.2 AVF from multi-shell diffusion MRI data

**Fig. 4:**
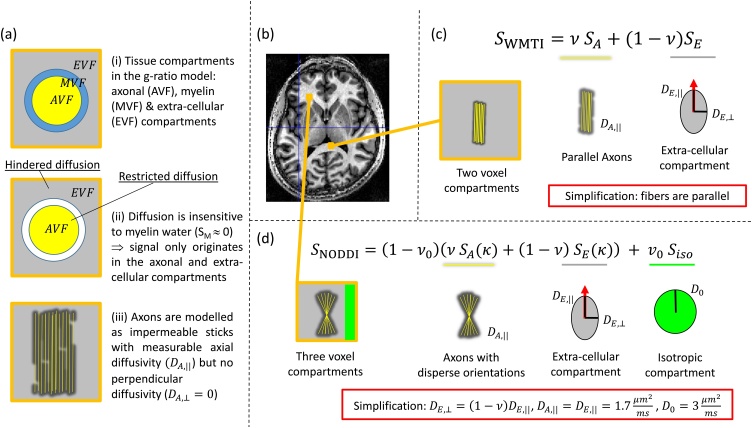
Depicted are the compartments of the g-ratio white matter (WM) tissue model as seen by diffusion MRI (a). An axial view of the human brain (b) is used to indicate WM regions where two example signal models that have been used to estimate the axonal water fraction (AWF) are applicable (c,d). (a.i): The cross-section of a representative myelinated axon and the associated tissue compartments in the g-ratio model: axonal (A in yellow), myelin (M in blue), and extra-cellular (E in gray) volume fractions (VF). (a.ii): Only two out of three compartments of (a.i) contribute to the diffusion signal: $S_{E}$ and $S_{A}$. The contribution from myelin is negligible because of its short T_2_, *i.e*. $S_{M}$ = 0. (a.iii): Typical diffusion models assume that the axonal compartment is composed of a population of sticks (depicted lengthwise in yellow) in which there is measureable diffusivity only along the length of the sticks (*i.e*. $D_{A,||}>0$ and $D_{A,⊥}\approx 0$). (c): The White Matter Tissue Integrity (WMTI) model is comprised of distinct signal contributions from within axons ($S_{A}$) and from the extra-cellular space ($S_{E}$) with corresponding signal fractions: ν and (1-ν) respectively as well as compartment-specific diffusivities: $D_{A,||}$, $D_{E,||}$ and $D_{E,⊥}$. However, WMTI can only be applied to WM regions with well-aligned fibre pathways because it assumes parallel sticks thereby excluding disperse fibre orientations. To satisfy this model assumption it has been used only within the corpus callosum (see (b)). (d) The Neurite and Orientation Dispersion in Diffusion Imaging (NODDI) model is comprised of axonal ($S_{A}$) extracellular ($S_{E}$) and isotropic ($S_{iso}$) signal compartments with signal fractions ν, (1-ν) and $\nu_{0}$ respectively. To improve fitting stability, the NODDI model makes very strong assumptions, e.g.: the intra- ($D_{A,||}$) and extra-axonal parallel diffusivities ($D_{E,||}$) are assumed to be the same and are fixed, as is the diffusivity of the isotropic compartment ($D_{0}$) corresponding to CSF. The parallel and perpendicular diffusivities are assumed to be related *via* the tortuosity model: $D_{E,⊥}=D_{E,||}(1-\nu )$. The depicted values are for the healthy *in vivo* case. However, NODDI does not assume parallel fibres, but rather accounts for fibre dispersion (κ), which is described by a Watson distribution (Stoyan, 1988). NODDI can therefore be used in regions with more disperse fibre orientations (as depicted in (b)).

Using a more extensive set of experimental measurements, *i.e*. multiple b-values or diffusion shells, allows the second category of studies to use a more principled model for the diffusion signal, the so-called “standard model” (Novikov et al., 2019). The standard model is built upon well-established signal models for two tissue compartments (for a summary see, e.g., (Novikov et al., 2019)), the axonal (AVF) and extra-cellular (EVF) volume fractions (Fig. 4a.ii). A restricted signal component is assumed to come from the axonal compartment, which is modelled as impermeable sticks (Fig. 4a.iii). A hindered signal component describes the extra-cellular space, which is modelled using a 3D anisotropic diffusion tensor. For example, the White Matter Tissue Integrity (WMTI) model is depicted in Fig. 4c showing the axially-symmetric ellipsoidal tensor composed of axial ($D_{\text{E},||}$) and perpendicular ($D_{\text{E},⊥}$) extra-cellular diffusivities.

In contrast to g-ratio studies based on DTI data, those using multi-shell diffusion MRI data acknowledge the fact that the direct contribution of myelin water in the diffusion MRI signal is negligible (Fig. 4a.ii). As a consequence, their models take into account that the axonal compartment estimated from the visible MRI signal in a typical diffusion experiment is not $AVF=\frac{AVF}{MVF+AVF+EVF}$ (Fig. 4a.i) but rather the axon water fraction (AWF), *i.e*. intra-axonal signal divided by the signal from the extra- and intra-cellular space: $AWF=\frac{f_{\text{A}\text{W}}}{f_{\text{A}\text{W}}+f_{\text{E}\text{W}}}$ (Fig. 2d) and thus $AWF=\frac{AVF}{AVF+EVF}$ (Fig. 4a.ii). These studies follow the suggestion of Stikov et al. (Stikov et al., 2015) to estimate AVF by rescaling the AWF accounting for the unsampled MVF, *i.e*.:

(2) $$AVF=\left(1-MVF\right)\;AWF$$

This rescaling inherently assumes that the entire bound (*i.e*. MR invisible) pool is associated with myelin, *i.e*. that $f_{\text{B}\text{N}\text{M}}=0$.

*WMTI:* The WMTI model (Fieremans et al., 2011) contains signal contributions from intra-axonal ($S_{\text{A}}$) and extra-cellular ($S_{\text{E}}$) compartments in line with the “standard model”. The signal fraction of sticks ($\nu =\frac{f_{\text{A}\text{W}}}{f_{\text{A}\text{W}}+f_{\text{E}\text{W}}}$, Fig. 2d) is directly used as proxy for AWF while 1-ν ($=\frac{f_{\text{E}\text{W}}}{f_{\text{A}\text{W}}+f_{\text{E}\text{W}}}$, Fig. 2d) estimates the extra-cellular water fraction (Fig. 4c). WMTI simultaneously estimates AWF, the intra-axonal diffusivity ($D_{\text{A},||}$) and two extra-cellular diffusivities ($D_{\text{E},⊥}$ and $D_{\text{E},||}$) of an axially-symmetric ellipsoidal tensor. However, it assumes parallel fibres and therefore has only been applied to the corpus callosum (West et al., 2018a).

*mcSMT*: Like WMTI, the multi-compartment Spherical Mean Technique (mcSMT) model developed by Kaden et al. (Kaden et al., 2016) is based on the “standard model”. But, instead of assuming parallel fibres, it uses the SMT to factor out the contribution of fibre orientation. As a result, it can be applied to the whole brain. Similar to the WMTI model, mcSMT estimates the signal fraction of the intra-axonal space, ν. This has been used as a proxy for the AWF in g-ratio mapping (West et al., 2018a). In the mcSMT model, the intra- and extra-cellular parallel diffusivities are assumed to be equal ($D_{\text{A},||}=D_{\text{E},||}$) and the tortuosity model (Szafer et al., 1995) is used to relate the extra-cellular parallel and perpendicular diffusivities to each other *via* ν: $D_{\text{E},⊥}{=\left(1-\nu \right)D}_{\text{E},||}$.

*NODDI:* The most commonly used method to estimate the AWF in g-ratio mapping has been the NODDI model (Fig. 4d, (Zhang et al., 2012; Stikov et al., 2015)). NODDI extends the standard model to 3 compartments by not only modelling the two signal compartments from the intra-axonal and extra-cellular spaces but also an isotropic signal component ($S_{iso}$ with an associated signal fraction ${\text{ν}}_{0}=\frac{f_{\text{C}\text{S}\text{F}}}{f_{\text{A}\text{W}}+f_{\text{E}\text{W}}+f_{\text{C}\text{S}\text{F}}}$, Fig. 2d) to account for any partial-volume contamination by freely diffusing water, e.g., as in CSF. To compensate for the increased number of model parameters and stabilize model fitting, the diffusion constants are fixed (Fig. 4d). To this end, as in the mcSMT model, the tortuosity model is used to relate the extra-axonal diffusivities *via* ν: ($D_{\text{E},⊥}{=\left(1-\nu \right)D}_{\text{E},||}$). Moreover, the intra-axonal and extra-axonal parallel diffusivities are assumed to be equal ($D_{\text{A},||}=D_{\text{E},||}$) and have a predefined value, as does the diffusivity of the isotropic compartment ($D_{0}$). To account for the 3-compartment nature of NODDI, Stikov et al. (2015) suggested the following relation between the NODDI signal fractions (Fig. 4d) and the $AWF:\;AWF=\nu \left(1-\nu_{0}\right).$ By scaling ν with $1-\nu_{0}$, ($\nu \left(1-\nu_{0}\right)=\frac{f_{\text{A}\text{W}}}{f_{\text{A}\text{W}}+f_{\text{E}\text{W}}}\left(1-\frac{f_{\text{C}\text{S}\text{F}}}{f_{\text{A}\text{W}}+f_{\text{E}\text{W}}+f_{\text{C}\text{S}\text{F}}}\right)=\frac{f_{\text{A}\text{W}}}{f_{\text{A}\text{W}}+f_{\text{E}\text{W}}+f_{\text{C}\text{S}\text{F}}}$) such that the intra-axonal signal fraction is corrected for the contribution of the CSF compartment, to ensure the g-ratio WM model assumption, *i.e*. AVF+MVF+EVF=1.

NODDI accounts for fibre dispersion using the single-parameter Watson distribution (Stoyan, 1988; Jespersen et al., 2012), making it applicable for whole brain AVF mapping.

*CHARMED:* Compared to other diffusion models that have been used for g-ratio mapping, the Combined Hindered and Restricted Models of water diffusion (CHARMED) approach makes the fewest assumptions. It models diffusion in the extra-cellular space by a full ellipsoidal tensor (whereas the NODDI and WMTI models assume an axially-symmetric ellipsoid), and, in principle, it can account for crossing fibre configurations (Assaf et al., 2004; Assaf and Basser, 2005) unlike the standard NODDI approach. The CHARMED model can be further extended to additionally estimate axon diameters (e.g. (Assaf et al., 2008; Alexander et al., 2010; Huang et al., 2016)). This has been used by Duval et al. for g-ratio mapping in the spinal cord (Duval et al., 2017) and by Yu et al. (Yu et al., 2019) in patients with multiple sclerosis. However, such a protocol requires more extensive (and time-consuming) data acquisition.

#### 2.3.3 Protocols for AVF mapping

While the first category of studies requires only a standard single-shell DTI protocol (Stikov et al., 2011; Mohammadi et al., 2015; Berman et al., 2018), the minimum requirement protocol for the second category of studies depends on the model to be used for AWF mapping. The WMTI model parameters can be estimated from the diffusion kurtosis tensor measurement (Fieremans et al., 2011; Jespersen et al., 2018). The NODDI, mcSMT, and WMTI model parameters can be estimated from a two-shell diffusion MRI protocol composed of a “lower” ($b∼1\frac{ms}{\mu m^{2}}$) and a “higher” diffusion weighting ($b∼2\frac{ms}{\mu m^{2}}$)^1^ . In contrast to the aforementioned models, the CHARMED model typically requires a more extended diffusion MRI protocol: Drakesmith et al. used a five shell diffusion MRI dataset for g-ratio weighted imaging (Drakesmith et al., 2019a). Extending the CHARMED model to also estimate axon diameters requires an even more advanced protocol where the b-values and additional diffusion parameters such as diffusion sensitization times also have to be changed (see (Duval et al., 2017) for g-ratio mapping).

Typical protocol-associated issues that can introduce biases are: ceiling effects (*i.e*. ν=1) in white matter, which can be encountered with NODDI if b-shells are sub-optimally sampled (recommendations for optimal sampling are provided in (Zhang et al., 2012)). Rician bias in low SNR data can also distort AWF estimates. Mapping accurate AWF parameters in the spinal cord comes with additional challenges because of increased susceptibility to nonlinear motion (e.g. due to swallowing, (Yiannakas et al., 2012)), physiological noise (e.g. (David et al., 2017)), or partial volume effects due to its small size (1 cm in diameter).

## 3 Challenges for aggregated g-ratio mapping

An important prerequisite of g-ratio mapping with MRI is that the biomarkers of MVF and AVF be accurate. Two key requirements for an accurate biomarker are model validity and a one-to-one correspondence between the MRI-biomarker and the gold standard volume fractions. While the first point can be investigated by theoretical evaluation of the model, the second point is typically not fulfilled, necessitating a calibration step. Another important challenge is related to imaging artefacts and their impact on the multi-modal combination of MVF and AVF biomarkers. In this section, we will first discuss the question of model validity associated with MRI-based MVF and AVF biomarkers, then we will use a simulation experiment based on *ex vivo* data to improve our understanding of the calibration step, and finally we discuss imaging artefacts associated with the multi-modal combination of MRI data.

### 3.1 Model validity

It is important to bear in mind that “all models are wrong but some are useful”^2^ . In the following sections we will cover some of the key model assumptions made to facilitate *in vivo* mapping of the AVF and MVF and enable g-ratio mapping. We will also discuss the consequent limitations of application. We focus solely on white matter for which the presented g-ratio models have been developed.

#### 3.1.1 MVF models

The simplest model for estimating $f_{B}$ is based on PD mapping, in which a mono-exponential, *i.e*. single water compartment, is typically assumed when extrapolating the signal to a TE of 0 ms to remove confounding T_2_^(*)^ decay. This is clearly not valid and constituent water compartments within a voxel will have variable influence depending on the echo times and spacings used (Whittall et al., 1999; Wiggermann et al., 2020). This will be the case for both PD mapping and MWI. In general, longer apparent T_2_^(*)^, and smaller fractional contribution from short T_2_ components, are observed as the first TE is increased or SNR lowers (Cercignani et al., 2018; Wiggermann et al., 2020). It is also important to fully sample the decay, which requires sufficiently long echo times to capture any slowly decaying compartments, e.g. CSF. See Section 2.2.3 and Table A1 (in Appendix A) for further details on protocol considerations.

Moreover, it has recently been shown that MWF depends on iron content (Birkl et al., 2019), the orientation of fibres with respect to the external magnetic field and on the TR used (Birkl et al., 2020) and exact processing details (Wiggermann et al., 2020). Sensitivity to B_0_ inhomogeneity can also bias model fits as can phase errors caused by physiological effects, such as breathing, eddy currents (Nam et al., 2015a) and motion, which distorts the decay (Magerkurth et al., 2011). Vulnerability to physiology and motion, together with partial volume effects, are particularly problematic for spinal cord imaging (Duval et al., 2017, 2018; Hori et al., 2018). More generally, these potential sources of artefact can manifest differently *in vivo* and *ex vivo*, meaning that while some techniques may work well in post mortem data, e.g. achieving cross-validation with histological data, they may not necessarily work well *in vivo*.

Models assuming two pools, either distinct non-exchanging water pools in myelin water imaging (Fig. 2b) or a bound and a free pool that interact *via* magnetisation transfer (Fig. 2c), are also limited by the fact that they do not describe the full complexity of the tissue’s microstructure. Higher numbers of pools are undoubtedly present (c.f. even the simplified model of Fig. 2a) but are unlikely to be distinguishable based on observable relaxation behaviour either because of exchange conditions or because it would require unattainable measurement precision. Simulation studies of more complete models have helped us to better understand the limitations of these simplifications.

In MWI, a slow exchange rate is central to the possibility of differentiating water pools, and their fractional sizes, based on experimentally distinguishable T_2_ times. As the exchange rate increases to a more intermediate regime, distinct compartments may still be discernible, but the relaxation times will appear reduced, as will the MWF (Does, 2018). The situation is further complicated by the presence of noise, which, even at low levels, can further broaden the distribution of apparent relaxation times, and lead to distinct water environments merging in the three pool case (Does, 2018).

The rate of magnetisation transfer exchange between macromolecular and water pools is an order of magnitude larger than the diffusion-driven exchange rate between water compartments (c.f. non-directional exchange rates of 10s^−1^ and 100s^−1^ respectively, (Levesque and Pike, 2009)). Theoretical analysis of a four pool model (analogous to Fig. 2a) has also shown that inter-compartmental exchange could substantially alter the estimated MWF, but that the qMT-based BPF is more robust (Levesque and Pike, 2009).

In support of these theoretical analyses, much greater variation in MWF than BPF has been seen in the spinal cord, not only *ex vivo* (Dula et al., 2010) but also *in vivo* (Harkins et al., 2012). The variability observed across tracts was consistent with variable exchange due to differences in axon diameter and myelin thickness, the key determinants of the g-ratio. Much of the extensive validation work for the MWI technique has been conducted *ex vivo*, and often with samples at room temperatures. Both of these factors serve to slow the rate of exchange increasing the validity of the slow exchange assumption (Does, 2018). Therefore, one must exercise caution extrapolating the validity of MWF metrics from *ex vivo* findings to the *in vivo* situation.

Although these three and four pool models are likely to be closer to the true tissue microarchitecture, inversion of such a complex model would be difficult in terms of both precision and bias. Indeed, even in the context of the two pool models that have been used to date for g-ratio mapping, the parameterisation must be supported by the data. The comparatively high parameterisation of the mcDESPOT model has necessitated the use of advanced fitting procedures, such as stochastic genetic or region contraction algorithms (Deoni et al., 2008, 2013). The achievable precision and accuracy of the approach has been called into question (Lankford and Does, 2013; West et al., 2019) and it has been shown to suffer from degeneracy when seeking to determine optimal model parameters, which is only resolved by using a simpler model, excluding exchange (West et al., 2019). A common requirement of all model types, including those capturing the AWF, is that any fixed parameters, e.g. as might be assumed in qMT models where the T_1_ of the free and bound pools may be assumed to be equal (Cabana et al., 2015), be appropriate to the population under consideration be they adults, children or indeed patients.

While it is also incorrect to assume that the non-aqueous compartment of tissue is entirely comprised of myelin, this has been shown to be the dominant source of the MT contrast mechanism in WM (Eng et al., 1991). In reality, the bound pool, $f_{\text{B}}$, can be associated not only with the lipids and proteins of the myelin sheath, $f_{\text{B}\text{M}}$, but also with any other macromolecule-bound protons, $f_{\text{B}\text{N}\text{M}}$ (see Fig. 2a), e.g. glial cells (MacKay and Laule, 2016).

MWF will not only capture water within myelin sheaths surrounding axons but also that associated with any myelin debris in pathological cases, as has been shown in peripheral nerve (Webb et al., 2003). Similarly, MT-based measures lack specificity. Hence it should be borne in mind that although alterations in myelin content will change the measured MT effect, an alteration in MT effects cannot be uniquely attributed to a change in myelin and may be driven by other macromolecular changes, or changes in T_1_ or T_2_. The derived MVF is also used to correct for the fact that the diffusion signal is insensitive to this compartment (by rescaling AWF). However, this neglects the non-myelin-macromolecular contribution within the imaging voxel, *i.e*. $f_{\text{B}\text{N}\text{M}}$ (Fig. 2a).

#### 3.1.2 AVF models

Diffusion MRI typically measures AWF and uses knowledge of the MVF to rescale it to the AVF (Section 2.3.2). As a result, the accuracy of both the AWF and MVF dictates that of the AVF. Examples of strong simplifications used by the AWF models are that the restricted compartment is solely associated with axons that can be modelled as impermeable sticks without cross-section, and that diffusion in the extra-cellular space is assumed to be Gaussian. The assumption that the restricted compartment is solely associated with axons is expected to be approximately correct in white matter if the density of other cells is small relative to the density of axons.

In addition to these model limitations, there is another problem associated with all of the approaches used for g-ratio mapping to date: they are based on the standard model comprised of compartments accounting for restricted and hindered diffusion. This model is known to suffer from a degeneracy of parameter estimates (Jelescu et al., 2016a) when measured with a linear diffusion weighting approach, *i.e*. the typical Stejskal and Tanner (Stejskal and Tanner, 1965) diffusion weighting scheme, which has been the case for all the aforementioned g-ratio mapping studies.

Prior assumptions motivated by the biological composition of the tissue can be imposed to stabilize the parameter estimation. The NODDI, mcSMT, and WMTI models make particularly strong use of priors to allow the remaining model parameters to be estimated from data that can be acquired in a clinically feasible imaging time (see Section 2.3.3). Parameter estimation is commonly stabilized by imposing the tortuosity assumption (Szafer et al., 1995), as is the case for both the NODDI and mcSMT models. This assumption constrains the perpendicular and parallel extra-axonal diffusivities *via* “one minus the neurite density”: ($D_{E,⊥}{=\left(1-\nu \right)D}_{E,||}$), *i.e*. the higher the neurite density in the tissue the lower the perpendicular diffusivity. However, the validity of this tortuosity constraint in densely packed axons has been questioned (Jelescu and Budde, 2017). Common to all models is the fact that they are measuring signal fractions, which are not corrected for potentially different T2 relaxation times, e.g., in the intra- and extra-axonal water (Veraart et al., 2018; Lampinen et al., 2019; McKinnon and Jensen, 2019; Gong et al., 2020). If the tortuosity constraint were indeed valid, it should relate extra-axonal diffusivities to the extra-cellular space (EVF=1-FVF) rather than signal fraction of the hindered compartment, ν (Jelescu et al., 2015). In other words, the relationship between the parallel and perpendicular extra-cellular diffusivities should effectively be: $D_{E,⊥}{=\left(1-FVF\right)D}_{E,||}$.

NODDI and mcSMT also impose a one-to-one scaling between the intra- and extra-cellular parallel diffusivities: ${D_{A,||}=D}_{E,||}$. The difference between NODDI and mcSMT (and WMTI) centres on the additional compartment that is estimated in NODDI ($\nu_{0}$). To facilitate the estimation of $\nu_{0}$, NODDI fixes the remaining diffusivity to a constant value (for *in vivo* healthy adults the diffusivities are usually assumed to be (Alexander et al., 2010; Guerrero et al., 2019):$\;{D_{A,||}=D}_{E,||}=\frac{1.7\mu m^{2}}{ms}$ and $D_{0}=\frac{3\mu m^{2}}{ms}$), whereas mcSMT estimates it. Although, it can be advantageous to estimate $\nu_{0}$ in certain situations (e.g. when partial volume effects are expected), it comes at the price of fixing the diffusivities which might be problematic, e.g. in children, patients, or post mortem brains, where these fixed diffusivities may no longer hold. WMTI, on the other hand, does estimate these diffusivities but assumes that all fibres are aligned in parallel restricting its application to anatomical regions that better, though not fully, support this assumption, e.g. the corpus callosum (West et al., 2018a). This might be another reason (in addition to fixed diffusivities used in NODDI) for the systematically smaller AWF estimates obtained with WMTI compared to NODDI as reported, e.g., in (Jelescu et al., 2015).

The Watson distribution used in NODDI can model fibre dispersion in a single fibre population, but cannot describe more complex fibre scenarios, such as crossing fibres. Nevertheless, it accounts, to a certain degree, for the variability of fibre-alignment within fibre pathways and thus might be better suited for g-ratio mapping across the entire white matter than models that assume strictly parallel fibre configurations.

Of course this list of model assumptions is not exhaustive. Additional considerations are discussed elsewhere (Jelescu and Budde, 2017; Novikov et al., 2019).

### 3.2 Calibration for MVF

**Fig. 5:**
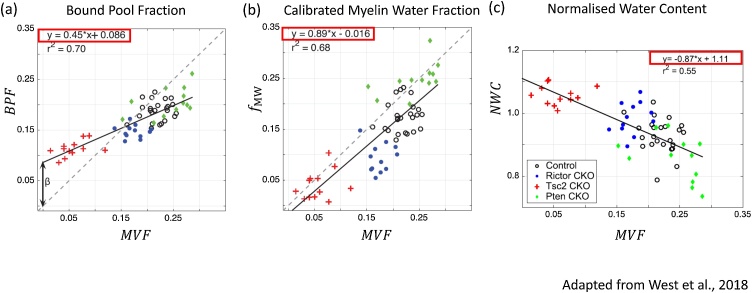
Depicted are the linear relations between the myelin-volume fraction (MVF) from gold standard electron microscopy and three MRI-based biomarkers for myelin: (a) Bound Pool Fraction (BPF) from quantitative magnetization transfer imaging, (b) calibrated Myelin Water Fraction ($f_{\text{M}\text{W}}$) from myelin water imaging, (c) the Normalized Water Content (NWC). The estimated linear relations (red boxes) are used in our simulation experiment (see Section 3.2 & Appendix). Macromolecular Tissue Volume (MTV) was calculated from NWC according to Berman et al. (Berman et al., 2018). Modified and reproduced with permission from West et al. (West et al., 2018b).

Assuming that the diffusion-based AWF is accurate^3^, the relation between the myelin biomarker and the MVF still needs to be established *via* a calibration step. This calibration is particularly important since it is not only required to quantify the MVF, but also to convert the AWF to AVF (Eq. 2). Histological investigations suggest that the relationship between typical myelin biomarkers (which we will collectively denote $M_{\text{M}\text{R}\text{I}}$ in this section) and the MVF is linear (Fig. 5, (West et al., 2018b)):

(3) $$MVF_{\text{M}\text{R}\text{I}}=\alpha M_{\text{M}\text{R}\text{I}}+\beta$$

where α and β are unknown coefficients that need to be calibrated. It is expected that these coefficients will depend on instrumental variables and may therefore vary with MR systems, sequence parameters, as well as myelin biomarker models. For example, B1^+^ inhomogeneity increases with field strength, and may lead to system-dependent residual differences and therefore different βs. Such dependency clearly limits the reproducibility and comparability of the MR-based g-ratio. Using simulations, Campbell et al. (2018) demonstrated that imperfect calibration can not only introduce a bias in the g-ratio, but can even cause the g-ratio to depend on the fibre volume fraction, negating a major strength of the g-ratio, *i.e*. that it is independent of FVF. Their simulations revealed that this dependence was different if the miscalibration was present only in the offset or only in the slope. They coined the phrase *aggregated g-ratio weighted imaging* to acknowledge this limitation (Campbell et al., 2018a).

To reduce these dependencies, two calibration methods have been used for *in vivo* g-ratio mapping. These have utilised a region of interest (ROI) in which either (a) the myelin biomarker was calibrated against a reference MVF, first employed by (Stikov et al., 2015) or (b) the measured g-ratio was calibrated against a reference g-ratio, first employed by (Mohammadi et al., 2015). We refer to these approaches collectively as ***single-point calibration*** methods since both are calibrating against a single reference value. Reformulating Eq. 3 within a specific ROI, it is clear that the single-point calibration methods estimate one effective proportionality constant ($\alpha_{\text{e}\text{f}\text{f}}$), *i.e*.:

(4) $$MVF_{\text{M}\text{R}\text{I}}(ROI,\alpha_{\text{e}\text{f}\text{f}})=\left(\alpha +\frac{\beta }{M_{\text{M}\text{R}\text{I}}\left(ROI\right)}\right)M_{\text{M}\text{R}\text{I}}\left(ROI\right)\equiv \alpha_{\text{e}\text{f}\text{f}}(\alpha ,\beta ,M_{\text{M}\text{R}\text{I}})\;M_{\text{M}\text{R}\text{I}}(ROI)$$

From Eq. (4) it is clear that the single-point calibration methods are insufficient to establish a one-to-one correspondence between the MVF and the MRI-based myelin biomarker. One problem, for example, could be that $\alpha_{\text{e}\text{f}\text{f}}$ will depend on the myelin biomarker within the reference ROI if β≠0 (see Eq. (4)). The MVF-based single-point calibration method would simply set Eq. 4 to a reference MVF value within the ROI: $MVF_{\text{M}\text{R}\text{I}}(ROI,\alpha_{\text{e}\text{f}\text{f},\text{o}\text{p}\text{t}})$ = $MVF_{\text{R}\text{E}\text{F}}$ and rearrange the equation with respect to $\alpha_{\text{e}\text{f}\text{f},\;\text{o}\text{p}\text{t}}$. The g-ratio based single-point calibration would minimize the following equation:

(5) $$\alpha_{\text{e}\text{f}\text{f},\text{o}\text{p}\text{t}}=\underset{\alpha_{\text{eff}}}{\text{min}}\left\|g\left(\alpha_{\text{e}\text{f}\text{f}},ROI\right)-g_{\text{R}\text{E}\text{F}}(ROI)\right\|$$

with $g\left(\alpha_{\text{e}\text{f}\text{f}},ROI\right)=\sqrt{1-\frac{MVF_{\text{M}\text{R}\text{I}}\left(ROI,\alpha_{\text{e}\text{f}\text{f}}\right)}{MVF\left(ROI,\alpha_{\text{e}\text{f}\text{f}}\right)+\left(1-MVF\left(ROI,\alpha_{\text{e}\text{f}\text{f}}\right)\right)AWF\left(ROI\right)}}$ where $g_{\text{R}\text{E}\text{F}}(ROI)$ and AWF(ROI) are the reference g-ratio and the measured AWF values within the ROI respectively.

The key questions that ensue from this single-point calibration are: what are the typical magnitudes of the slope α and offset β in experimental conditions and therefore what is the magnitude of the error propagated by $\alpha_{\text{e}\text{f}\text{f},\;\text{o}\text{p}\text{t}}$? How much does the MR-based g-ratio deviate from the ground truth? How large is this deviation relative to the expected dynamic range of the g-ratio, e.g. pathology-related differences?

Although the simulations in (Campbell et al., 2018a) improved our understanding of the pitfalls of g-ratio mapping, they did not directly answer these questions. However, experimental data from the Does lab (Kelm et al., 2016; West et al., 2018b, 2018a) could help to now answer them. In those experiments, the authors reported the changes of the g-ratio and the associated MVF in a range of mouse models spanning hypo- to hyper-myelination using both MRI and electron microscopy. The MRI based data included three biomarkers of myelin content: MWF ($f_{\text{M}\text{W}}$), BPF, and MTV. Since in this case MTV was derived from the MWI experiment (*i.e*. with a multi-compartment model) we denote it ${MTV}_{\text{M}\text{W}\text{I}}$. In the following, we will use the data from the Does lab to generate ground truth parameters for a subsequent simulation-based experiment to probe the potential and pitfalls of single-point calibration (details of which can be found in the appendix and supplementary material). Note that in this simulation experiment we only focus on the myelin proxies assuming that there is no error in the AVF measurement.

**Fig. 6:**
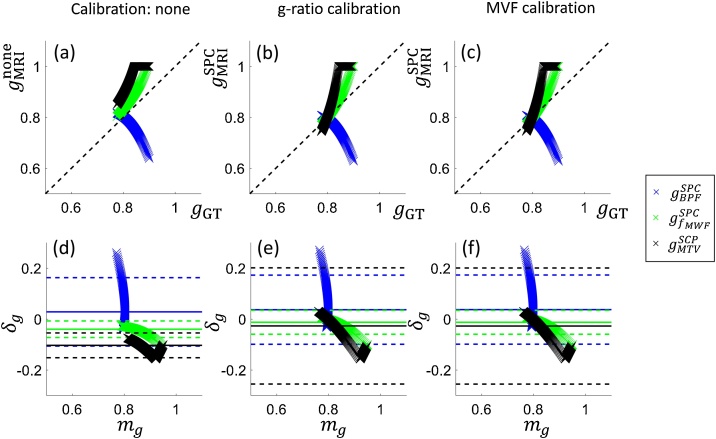
Depicted are scatter (a–c) and Bland-Altmann plots (d–f) of the ground truth ($g_{GT}$) and the MRI-based g-ratios using no calibration (a,d) or the single-point calibration approaches based on the g-ratio (b,e) or MVF (c,f) from a reference region of interest. The MRI-based g-ratios were calculated using different biomarkers for myelin: bound pool fraction (BPF,blue crosses), calibrated myelin-water fraction ($f_{\text{M}\text{W}}$, green crosses), and macromolecular tissue volume (MTV, black crosses). The Bland-Altman plots (Bland and Altman, 1986) assess the bias and error when seeking to replace the ground truth g-ratio with the MRI-based measures. The plots depict the difference (${{\delta_{g}=g}_{\text{G}\text{T}}-g}_{\text{M}\text{R}\text{I}}$) against the mean (${{m_{g}=(g}_{\text{G}\text{T}}+g}_{\text{M}\text{R}\text{I}})/2$) of g-ratios with the solid line indicating the mean difference $\langle \delta_{g}\rangle$, and the dashed lines indicating $\langle \delta_{g}\rangle$ plus/minus 1.96 times the standard deviation of the differences $\langle std_{{\text{δ}}_{\text{g}}}\rangle$ to encompass 95 % of the normal distribution. The results are summarized in Table A1 and in Appendix A.

We will evaluate the difference between the ground truth g-ratio, $g_{\text{G}\text{T}}$, and that obtained by simulated MRI measures, $g_{\text{M}\text{R}\text{I}}$. To do so, we will use bias and error as determined with Bland-Altman analyses (Bland and Altman, 1986). The Bland-Altman plots (Fig. 6) depict the difference (${{\delta_{\text{g}}=g}_{\text{G}\text{T}}-g}_{\text{M}\text{R}\text{I}}$) between the g-ratios as a function of their mean (${{m_{\text{g}}=(g}_{\text{G}\text{T}}+g}_{\text{M}\text{R}\text{I}})/2$). According to their original publication (Bland and Altman, 1986), mean difference $\langle \delta_{\text{g}}\rangle$ and $+/-1.96\;\langle {std}_{{\text{δ}}_{\text{g}}}\rangle$ can respectively be interpreted as the bias and error that would result if replacing $g_{\text{G}\text{T}}$ with $g_{\text{M}\text{R}\text{I}}$. ***Bias*** captures the offset from the ground truth g-ratio value, whereas ***error*** captures the deviation from a one-to-one relationship between the ground truth and the MR g-ratio. While a potential bias can be retrospectively corrected, any error in the g-ratio mapping method will define its sensitivity and ability to detect change or differences between individuals, groups or over time. Any error must be lower than the expected difference between groups or due to pathology if the g-ratio mapping method using MRI is to be of use as a reliable biomarker (Alberich-Bayarri et al., 2020).

#### 3.2.1 What we can learn from the simulation experiment

Overall, this simulation showed (Fig. 6, Table A1 (in Appendix A)) that the single-point calibration can reduce the bias in the g-ratio (*i.e*. two out of three MRI-based g-ratio values became, on average, closer to the ground truth) but it comes at the cost of an increased error (*i.e*. the deviation from a one-to-one correspondence between the MR and the ground truth g-ratio increased after calibration, particularly for those two with decreased bias). We expect that the latter feature, *i.e*. the error, is of more relevance to typical g-ratio studies, where longitudinal changes or changes between groups will likely be investigated. In more detail, the simulations showed that $f_{\text{M}\text{W}}$ and $MTV_{\text{M}\text{W}\text{I}}$ are better biomarkers for the g-ratio in terms of their error. Perhaps surprisingly, they perform best, in terms of error, when no calibration was performed. BPF, on the other hand, performed poorly as an MVF biomarker independent of whether or not a calibration was performed. Interestingly, the two better performing MVF biomarkers, *i.e*. $f_{\text{M}\text{W}}$ and $MTV_{\text{M}\text{W}\text{I}}$, involved a calibration step in their computation, unlike the BPF. For $f_{\text{M}\text{W}}$ the calibration was purely based on literature values, whereas $MTV_{\text{M}\text{W}\text{I}}$ was calibrated against a grey matter value specific to each brain.

Based on these simulations, a number of conclusions can be drawn. First, the single-point calibration method is insufficient to calibrate the g-ratio for the investigated scenarios with non-zero offset parameter. The impact of the calibration will depend on the specific markers sensitivity to myelin and other quantities (*i.e*. the slope and offset, Eq. 3). Second, of the particular markers investigated here the BPF-based g-ratio would require a more sophisticated calibration. On the other hand, the $f_{\text{M}\text{W}}$ and $MTV_{\text{M}\text{W}\text{I}}$ based measures could in fact be used without even a single-point calibration with the knowledge that this trades larger bias for sensitivity.

**Fig. 7:**
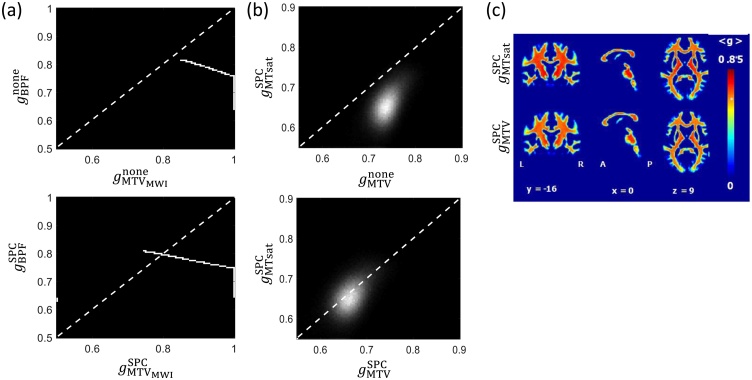
Illustration of the inter-relation between MR g-ratios derived from magnetisation transfer imaging (a: simulated ${\text{g}}_{\text{B}\text{P}\text{F}}$) and (b,c: *in vivo*$g_{\text{M}\text{T}}$) or from the macromolecular tissue volume (a: simulated $g_{\text{M}\text{T}\text{V}}$) and (b,c: *in vivo*$g_{\text{M}\text{T}\text{V}}$) for two scenarios: omitting ($g^{\text{n}\text{o}\text{n}\text{e}}$) or using single-point calibration ($g^{\text{S}\text{P}\text{C}}$) with a reference of $g_{\text{R}\text{E}\text{F}}=0.71$ in the medullary pyramid, estimated from (Graf von Keyserlingk and Schramm, 1984). The dynamic ranges of the MR g-ratios observed in simulation *ex vivo* (a) or *via in vivo* measurement (b,c) are very different. In both cases (a,b), there is a shift towards the identity line after SPC, but with much greater agreement between the measures *in vivo*. Note that the MTsat-based g-ratio is undefined without calibration because the range of MTsat within white matter exceeds 1. The maps in (c), adapted with permission from (Ellerbrock and Mohammadi, 2018a), were acquired using the protocol described in the caption of Fig. 8. Note that the MR g-ratios (“g3” and “g4”) in the original publication were erroneous due to a reported mistake, see corrigendum (Ellerbrock and Mohammadi, 2018b). Here, the correct maps are depicted.

While the key take home message is that the impact of the calibration will depend on the sensitivity of the marker to the underlying MVF, care must be exercised in extrapolating the specific findings to corresponding *in vivo* measures of BPF, MTV and MWF. The use of *ex vivo* data was necessary for this simulation experiment due to the lack of gold standard information *in vivo*. However, myelin markers can be expected to have different dependence on the MVF when measured *in vivo* in humans than seen here in the case of fixed tissue from *ex vivo* mice. Indeed, the scatter plots in Fig. 7, which depict the g-ratio estimates before^4^ and after calibration obtained *via* simulation (Fig. 7a) and from in *vivo* experiments using MTsat and MTV (Fig. 7b), do not manifest the same relationship. The MTsat-based *in vivo* g-ratio map, in fact, shows a greater dynamic range and higher correspondence to the MTV-based *in vivo* g-ratio map after single point calibration (Fig. 7b and c). This contrasting observation might be due to the use of somewhat different techniques *in vivo* and *ex vivo*, or due to fixation issues, e.g. fixation has been shown to strongly increase the BPF in normal appearing white matter (Schmierer et al., 2008). Additional important differences are potentially different model validity (see Section 3.1.1) and data quality, most notably the absence of physiological and motion noise sources *ex vivo*, the capacity for markedly longer scanning protocols, and the use of different MRI techniques and non-clinical imaging systems (West et al., 2018b). It should also be noted that the simulations assumed (1) no noise, (2) that the reference values for the g-ratio or MVF have no bias (3) knowledge of the true AVF, not AWF, and (4) modelled the specific case of the g-ratio changing due to demyelination.

In summary, these simulations show that the single-point calibration, used in virtually all *in vivo* g-ratio mapping studies to date (Table 1), does not fully resolve the issue of converting MR proxies to the true MVF and can even increase bias and error in the g-ratio estimates. Therefore, further methodological development and validation is required to find the optimal means of ensuring the necessary validity and sensitivity of the MR g-ratio.

### 3.3 Unification of multi-modal data

**Fig. 8:**
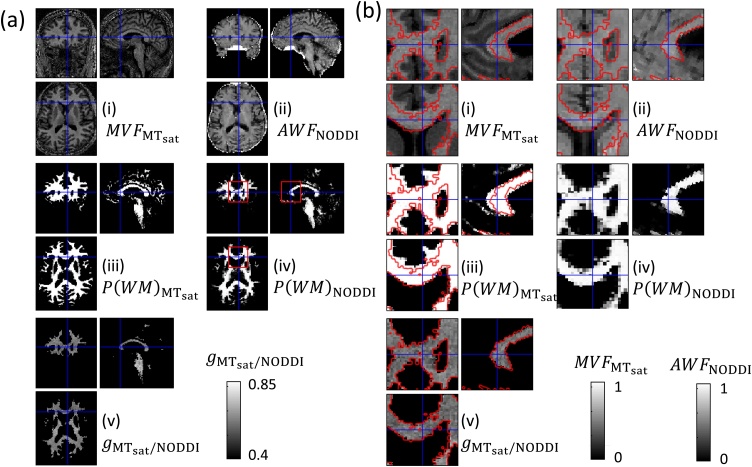
Depicted are whole brain views (a) (and magnifications (b)) of the aggregated g-ratio weighted map ((v): $g_{\text{M}{\text{T}}_{\text{s}\text{a}\text{t}}/\text{N}\text{O}\text{D}\text{D}\text{I}}$), its constituting two qMRI maps: the calibrated myelin biomarker ((i): $MT_{\text{s}\text{a}\text{t}}$), the axonal-water fraction ((ii): AWF), and the associated white-matter tissue probability maps (P(WM)) after their respective segmentation ((iii): ${\text{P}(\text{W}\text{M})}_{\text{M}\text{T}}$), (iv): ${\text{P}(\text{W}\text{M})}_{\text{A}\text{W}\text{F}}$). Although, a distortion-corrected AWF maps with negligible residual distortions was used, ${\text{P}(\text{W}\text{M})}_{\text{A}\text{W}\text{F}}$ is lower than the ${\text{P}(\text{W}\text{M})}_{\text{M}\text{T}}$ (see contours in the magnification (b)). To prevent artefactual g-ratio values in regions where one of the two constituent biomarkers is ill-defined, we suggest generating g-ratio values only in voxels where both tissue probabilities exceed a pre-defined threshold (here 0.5). Details of the MPM protocol can be found in Streubel et al. (2020), Streubel et al. (2020); AWF was estimated from a 3 shell DWI (b-values: 500, 1000, 2500 mm/s2). The spatial distortions were reduced using the ACID toolbox (www.diffusiontools.com).

The aggregated g-ratio weighted imaging approach combines two complementary MRI contrasts, sensitive to the axonal-water and myelin volume fractions respectively. Given that each quantitative MRI technique is typically vulnerable to a specific set of artefacts, the combination of multiple data types needs to take care not to amplify these artefacts such that they obscure or corrupt the quantity of interest. For example, we have previously demonstrated that modality-specific spatial distortions, arising from inhomogeneous magnetic susceptibility distributions in the brain and around air cavities, can prevent voxel-wise spatial correspondence of the AWF and MVF proxies being achieved and lead to erroneous g-ratio estimates (Mohammadi et al., 2015). Even after correcting the susceptibility-induced distortions using dedicated tools (Ruthotto et al., 2012, 2013), residual misalignments between the EPI-based diffusion data and the MRI-biomarkers for MVF can persist. The most obvious reason for residual misalignment is, of course, insufficient susceptibility distortion correction, but partial-volume effects in the EPI-based diffusion data associated with the typically lower spatial resolution, the EPI-readout, and eddy current distortions can also lead to lower white-matter tissue probability in the diffusion data relative to the MTsat map (Fig. 8). Here, we suggest combining the overlap between two modality-specific white-matter tissue probability maps (TPMs) to remove regions in the resulting g-ratio maps (Fig. 8a.v and b.v) that do not overlap between the two MRI contrasts, *i.e*. the region outside the red contours in Fig. 8a.iii and b.iii. In the example of Fig. 8, the TPM was generated from the MTsat (Fig. 8a.iii and b.iii) and NODDI (Fig. 8a.iv and b.iv) map, respectively.

### 3.4 Validation of g-ratio mapping

*In vivo* validation of g-ratio mapping is highly desirable, but generally unfeasible. We therefore typically rely on *ex vivo* histology for validation. A number of differences between these two imaging scenarios have been highlighted in previous sections. Here we summarise key points pertinent to the *ex vivo* histology gold standard scenario. It is important to ensure that the axons, sampled with *ex vivo* histology, are representative of the ensemble of axons that have been measured with MRI. For example, electron microscopy typically samples 100–1000 axons (e.g. (Aboitiz et al., 1992; Liewald et al., 2014)) whereas a typical *in vivo* MRI voxel contains 100k-1000k axons. Moreover, one has to consider the change in tissue composition that occurs when going from the *in vivo* ***to the ex vivo situation***. In this case, the MRI signal and its parameters can significantly change due to, e.g. (i) autolysis (varying post-mortem interval (Shepherd et al., 2009)), (ii) fixation and the associated changes of cross-linking proteins, tissue shrinkage, and slowed diffusion processes (Schmierer et al., 2008; Shepherd et al., 2009), and (iii) temperature changes (Birkl et al., 2016). These changes affect diffusion (Dyrby et al., 2011) and other important MR parameters, such as T_2_, T_1_, and T_2_* (Streubel et al., 2019) and susceptibility (based on signal phase) contrasts. However, despite these changes, the most important MRI mechanisms (e.g. diffusion anisotropy and relaxation mechanisms) are still present after fixation (Roebroeck et al., 2008). Nonetheless, it is necessary to characterize these differences in MRI parameters to enable translation and interpretation across *in vivo* and *ex vivo* measurements.

#### 3.4.1 g-ratio

To date, only two studies have compared g-ratio measurements from *ex vivo* histology with MRI (Stikov et al., 2015; West et al., 2018a). Stikov et al. (2015) compared the g-ratio measured with *in vivo* MRI and *ex vivo* histology on a macaque monkey. West et al. (2018a) compared g-ratio maps based on the WMTI, mcSMT and NODDI models to the equivalent g-ratio measured using gold standard histology techniques in mouse models. All three methods showed a moderate linear correspondence. It is important to note that the fixed diffusivities of NODDI had to be adjusted empirically for the *ex vivo* data. Another interesting finding was that a simplified g-ratio model, in which the extra-axonal volume fraction was assumed to be zero, such that AVF = 1 – MVF, performed equally well to the above mentioned diffusion signal models. The conclusions from this finding could be quite radical, *i.e*. that it is not necessary to measure both diffusion MRI and myelin markers to estimate changes in g-ratio across a strongly myelinating process. However, again caution is required since the gold-standard g-ratio (measured by histology) did not account for the contribution of unmyelinated axons, which the MRI g-ratio is also expected to depend on. Finally, it is important to highlight that, to date, no human specimen has been used to validate the g-ratio. This, however, would be a crucial step in linking *ex vivo* histology with our target *in vivo* application, *i.e*. g-ratio mapping in the human brain.

#### 3.4.2 MVF

Here we discuss comparisons between myelin-sensitive MRI-based metrics and the gold standard MVF measured *via* histology that have been carried out in the context of g-ratio mapping. In early work, Stikov et al. compared the PSR estimated *via* MRI with the MVF estimated from electron microscopy in the corpus callosum of a macaque (Stikov et al., 2015). They did not find a significant relationship, perhaps due to limited myelin-related variance present in the data. Using mouse models spanning hypo- and hyper-myelinated conditions has allowed a broader variance in myelination to be investigated (West et al., 2018b). West et al. used this approach to explore the relationship between the histological MVF, again derived from electron microscopy, and MRI-based measures in the same animals made using both MWI and qMT techniques (West et al., 2018b). They demonstrated a linear correlation between the MVF and both the MWF (r = 0.81) and the BPF (r = 0.84). These metrics have shown similar correlations with an MR-derived MVF, though the exact degree of correlation depended on the details of the MVF calibration (Jung et al., 2018; West et al., 2018b). Berman et al. (Berman et al., 2018) used data from the same study to explore the dependence of the MTV on MVF. Unlike the typical quantification approach used *in vivo*, this *ex vivo* MTV measure was derived from a PD estimate obtained by extrapolating the MWI data to a TE of 0 ms. A linear dependence on MVF was also demonstrated for this *ex vivo* MTV measure (R^2^ = 0.74). While these *ex vivo* observations of linear dependence of myelin-sensitive MR metrics on MVF lend credence to the calibration approach investigated in Section 3.1, they nonetheless reinforce the need for calibration since none show an offset-free 1:1 relationship. The previously outlined caveats regarding the translation of the methods from *ex vivo* to *in vivo* experiments must also be borne in mind.

#### 3.4.3 AVF

Validation of AVF presents some distinct challenges. The AVF estimated from diffusion-based metrics is sensitive to the pool of myelinated axons but also influenced by the unmyelinated axons *via* AWF (Beaulieu and Allen, 1994a, 1994b; Beaulieu, 2009; Jones, 2010) and non-myelin macromolecules *via* the extra-cellular volume fraction (EVF), *i.e*. $AVF=\left(1-\left(MVF+EVF_{NM}\right)\right)AWF$. By contrast, gold standard EM-based assessment of volume fractions often focus on the myelinated axons only (Kelm et al., 2016; West et al., 2018b, 2018a; Zaimi et al., 2018; Tabarin et al., 2019). The myelin sheath provides protection against autolysis and acts as a contrast-enhancer for microscopy, making myelinated axons likely to be present and more easily detectible than unmyelinated axons (Olivares et al., 2001). In 2D EM, unmyelinated axons can also be confused with non-neuronal processes from cells like astrocytes or microglia. Ideally, a high-resolution microscopy approach combined with a neuron-specific stain, e.g. for neurofilaments, should be used to assess the AVF by encompassing all axons. (Jelescu et al., 2016b) compared MRI-based AWF with a histological counterpart (*via* Eq. (2)), including both myelinated axons and an estimate of unmyelinated axons, in mouse models with different degrees of myelination. They found a linear relation, though not a 1:1 correspondence. This is an indication that MRI-based AWF also needs to be calibrated.

## 4 Conclusion and outlook

This review provides methodological background for the MRI techniques pertinent to aggregate g-ratio weighted mapping with the aim of improving understanding of the currently used biomarkers, as well as providing insight into the potentials and particularly the pitfalls. G-ratio weighted mapping has the potential to achieve non-invasive mapping of this functionally-relevant microstructural parameter by utilising the strength of multi-contrast quantitative MRI and biophysical models (also known as *in vivo* histology using MRI (Weiskopf et al., 2015)). The main take-home messages of this review are that: (1) to fully benefit from the advantages of the aggregate g-ratio model, further work on a more appropriate calibration method is necessary to enable simultaneous estimation of both the slope and offset of the relationship between MRI markers and the true MVF; (2) more *ex vivo* histology gold standard measurements of human brain tissue are required to assess the typical range of MR g-ratio values that can be expected *in vivo*, (3) the quest to find the most appropriate MRI biomarkers for MVF and AVF for the *in vivo* situation is ongoing. In particular, there is currently a lack of validation studies for biomarkers of the AVF compartment using diffusion-based metrics. A major challenge here will be the estimation of the contribution to the AVF from unmyelinated axons (and cells potentially) *via* histology.

Other models that combine WMTI parameters and fibre dispersion (as defined by Watson distribution, e.g., in NODDI) (Jelescu et al., 2015; Jespersen et al., 2018) might have the potential to combine the sensitivity of WMTI to compartmental diffusivities with the less strict assumption about fibre alignment of the NODDI model. However, they suffer from model-inherent degeneracies (Jelescu et al., 2016a). One proposed solution to this degeneracy is to combine linear encoding schemes with planar or spherical diffusion sequences (Reisert et al., 2018; Coelho et al., 2019). A few studies have compared the diffusion anisotropy and intra-cellular signal fraction from linear diffusion weighting with planar diffusion weighting sequences: (Henriques et al., 2019) did this *ex vivo* in mice and (Mohammadi et al., 2017) did it *in vivo* in humans. However, these techniques have not yet been used for aggregated g-ratio weighted imaging. Another study has revealed a one-to-one correspondence between a simplified NODDI model and the mean diffusivity and fractional anisotropy as measured with DTI, dubbed NODDI-DTI (Edwards et al., 2017). NODDI-DTI might help to link the models of g-ratio mapping studies based on a standard DTI protocol to those models based on more advanced diffusion MRI protocols. However, NODDI-DTI has also not yet been applied to g-ratio mapping.

Future directions might also include the use of generative signal models that directly depend on the MR g-ratio (e.g., (Wharton and Bowtell, 2012, 2013; Papazoglou et al., 2019)) to allow its extraction (Tendler et al., 2015; Drakesmith et al., 2019b), or alternatively estimating the g-ratio from a multi-compartment GRE signal model (Thapaliya et al., 2018, 2020) or solely using diffusion MRI measurements (Jelescu et al., 2015; Novikov et al., 2019). A great advantage of these techniques is that they do not depend on combining two different MRI contrasts but can instead estimate the MR g-ratio directly from a single contrast. However, further investigations are required to test their validity. New approaches that promise greater specificity to myelin (e.g. ihMT (Varma et al., 2015; Ercan et al., 2018; Duhamel et al., 2019)) and intra-axonal (Shemesh et al., 2016) compartments may also improve our capacity to directly map the g-ratio in the human brain *in vivo*.

## Figures and Tables

**Table A1:** The relative bias and error introduced by single-point calibration with a reference g-ratio (3rd row) or MVF (4th row) as assessed by Bland-Altman analysis (Fig. 6) for three different myelin biomarkers: Bound Pool Fraction (BPF, 2nd column), calibrated Myelin Water Fraction ($f_{\text{M}\text{W}}$, 3rd column), and Macromolecular Tissue Volume (MTV, 4th column). The reference values are noted in the last column. Bias is defined as the mean difference $\langle \delta_{\text{g}}\rangle$ while error is defined as the interval between +/-$1.96\;\langle {std}_{\text{δ}}\rangle$ with $\delta_{\text{g}}=g_{\text{G}\text{T}}-g_{\text{M}\text{R}\text{I}}$. Here, bias and error are expressed as a percentage of the dynamic range of the ground truth g-ratios: $dyn_{\text{G}\text{T}}=\text{max}\left(g_{\text{G}\text{T}}\right)-\text{min}\left(g_{\text{G}\text{T}}\right)=0.124$.

Calibration	BPF		$f_{\text{M}\text{W}}$		$MTV_{\text{M}\text{W}\text{I}}$		Reference value
	Bias [%]	Error [%]	Bias [%]	Error [%]	Bias [%]	Error [%]	
none	23.0	55.1	-31.9	13.3	-82.7	20.0	
g-ratio	29.8	55.7	-9.9	19.3	-21.5	93.6	0.797
MVF	30.1	55.7	-10.0	19.3	-21.8	93.6	0.175

**Table A2:** This table summarizes limitations of MRI-based techniques that have been used for MVF and AWF (FVF) measurement in the context of g-ratio mapping.

	Limitations of the AWF estimation approach (LA)
LA.1	Assumes parallel fibres and thus can be applied only in regions where this assumption is not violated (typically it has been applied in the corpus callosum).
LA.2	The TFD (Reisert et al., 2013) has been assumed to be proportional to the fibre volume fraction because the model does not distinguish between fibre and axonal compartments. It relies on a tractography algorithm and thus inherits the associated limitations. G-ratios based on this method show a larger scan-rescan variability as compared to NODDI-based g-ratios (Ellerbrock and Mohammadi, 2018a).
LA.3	NODDI and mcSMT use the tortuosity model to relate the perpendicular extra-axonal diffusivity to the parallel extra-axonal diffusivity scaled by “one minus the neurite density”: ($D_{E,⊥}{=\left(1-\nu \right)D}_{E,||}$). Moreover, NODDI and mcSMT impose a one-to-one scaling between the intra- and extra-cellular parallel diffusivities: ${D_{A,||}=D}_{E,||}$.
LA.4	NODDI fixes all diffusivities to a constant value (for *in vivo* healthy adults the diffusivities are usually assumed to be:$\;{D_{A,||}=D}_{E,||}=\frac{1.7\mu m^{2}}{ms}$ and $D_{0}=\frac{3\mu m^{2}}{ms}$).
LA.5	The WMTI model assumes parallel fibres and thus can applied only in regions where this assumption is not violated (typically it has been applied in the corpus callosum, but whether the model assumptions are sufficiently met there is unclear).
LA.6	These studies provided not sufficient information to assess the specific implementation of the diffusion model.
	Limitation of the MVF estimation approach (LM)
LM.1	Requires a conversion factor to convert MRI-based myelin marker to the myelin volume fraction, which is done *via* histological data in different species. If this conversion factor is incorrect, the g-ratio will not be decoupled from the FVF (Stikov et al., 2015).
LM.2	MTsat depends not only on the bound pool fraction but also on the rate of exchange, k, between the bound and free pools. Moreover it is a semi-quantitative measure because it depends on the particular off-resonance pulse used in the sequence, most notably its power and offset frequency (Helms et al., 2008).
LM.3	Results in biased (over-)estimates (West et al., 2019) with artifactually high precision (West et al., 2019; Lankford and Does, 2013).
LM.4	Requires estimation of the proton density and therefore a normalisation factor, e.g. the proton density in CSF. The optimal choice of the normalisation region will depend on the acquisition scheme, and will dictate the precision and accuracy of the MTV estimate. The modulation of the receiver coil’s sensitivity also needs to be removed, either by constrained model fitting or measurement (Mezer et al., 2016).
LM.5	The MTR suffers from the same limitations as MTsat, but retains dependence on both T1 and transmit-field inhomogeneities. These additional dependencies make it more prone to error as demonstrated e.g. in (Callaghan et al., 2015).
LM.6	When fitting magnitude multi-echo data with long echo times, significant biases can be introduced by the Rician noise distribution that can greatly alter the measured T_2_(*) values (Bjarnason et al., 2013).
	Fitting results are sensitive to the choice of the number of echoes acquired, their TEs and the echo spacing, e.g. higher apparent T_2_(*) and smaller fractional contributions from short T_2_ species as the first echo is increased (Whittall et al., 1999; Cercignani et al., 2018). A broad range of echo times are required to fully characterise both long and short T2 components.
	Short echo times are required to acquire a signal with appreciable contribution from myelin, which is particularly problematic for gradient echo imaging due to the very short T_2_* of myelin.
LM.7	Error can result from the sensitivity to B1+ effects, both inhomogeneity, which can lead to stimulated echoes distorting the decay, and slice profile effects for 2D acquisitions (Lebel and Wilman, 2010). Power deposition can also be problematic, particularly at UHF.
LM.8	Sensitivity to B0 inhomogeneity can bias model fits (Nam et al., 2015a). Phase errors caused by breathing and eddy currents can also lead to errors if uncorrected (Nam et al., 2015b).
LM.9	Assumes a two pool model, which is a simplification, but likely sufficient to be supported by *in vivo* data acquired in the human brain (Levesque and Pike, 2009).
LM.10	The model validity is unknown.

**Table A3:** MRI Methodological Abbreviations.

Myelin Imaging Techniques		
MWI	Myelin Water Imaging	
MET2(*)	Multi-Exponential fitting to map compartment-specific T_2_(*)	
qMT	Quantitative Magnetisation Transfer	
bSSFP	Balanced Steady State Free Precession	
SPGR	SPoiled Gradient Recalled echo	
mcDESPOT	Multi-Compartment Driven Equilibrium Single Pulse Observation of T_1_ and T_2_	

Diffusion Imaging Techniques		
DWI	Diffusion Weighted Imaging	
DTI	Diffusion Tensor Imaging	
NODDI	Neurite Orientation and Dispersion Diffusing Imaging	
CHARMED	Composite Hindered And Restricted Model of Diffusion	
WMTI	White Matter Tissue Integrity	
TFD	Tract Fibre Density	
mcSMT	Multi-Compartment Spherical Mean Technique	

Biomarker and volume fractions		
BPF (f)	Bound pool fraction	Bound pool magnetisation relative to the combined bound and free pool magnetisation amplitudes as measured using qMT.
PSR (F)	Pool size ratio	Bound pool magnetisation relative to free pool magnetisation amplitude as measured using qMT.
AVF	Axonal volume fraction	The fraction of the imaging voxel volume that is intra-axonal.
AWF	Axonal water fraction	The fraction of the MRI water signal originating from the axonal compartment.
MVF	Myelin volume fraction	The fraction of the imaging voxel volume associated with myelin. This includes both the myelin itself and the water trapped between its bilayers.
MWF	Myelin water fraction	The fraction of the MRI water signal identified as exhibiting faster relaxation and attributed to the water trapped within the myelin sheath.
EVF	Extra cellular volume fraction	The fraction of the imaging voxel volume that originates outside the fibre.
FVF	Fibre volume fraction	The fraction of the imaging voxel volume that originates outside the fibre.
PD	Proton density	The concentration of MR-visible water relative to the concentration in the same volume comprised entirely of water.
MTV(F)	Macromolecular tissue volume (fraction)	The (fractional) volume of the imaging voxel that is comprised of macromolecules, *i.e*. that is not MR-visible water.
MTsat	Magnetisation transfer saturation	The steady state signal loss as a result of magnetisation transfer between the bound and free pools.

## Appendix A *Ex vivo* simulation experiment

### Methods

To generate a realistic range of ground truth values for the MVF and AVF, we fitted the histology-based MVF and AVF values reported in (West et al., 2018b, 2018a) using a heuristic relation between AVF and MVF (Fig. S1a, Eq. (S1)). Then, we used Eq. (S2) to generate the ground truth g-ratio values ($g_{\text{G}\text{T}}$, which ranged from 0.77 to 0.90, Fig. S1b).To generate the simulated $g_{\text{M}\text{R}\text{I}}$, we used: $g_{MRI}=\sqrt{1-\frac{M}{M+AVF_{\text{G}\text{T}}}}$ with $AVF_{\text{G}\text{T}}$ being the ground truth AVF and M being the MRI based myelin marker. To generate the MRI-based myelin marker, we used the linear relationships reported in (West et al., 2018b) between the histological MVF (here: the ground truth MVF) and three myelin biomarkers: BPF=0.45 MVF+0.086 (Fig. 5a^5^), $f_{\text{M}\text{W}}=0.89\;MVF-0.016$ (Fig. 5b), and $MTV_{\text{M}\text{W}\text{I}}=0.75\;MVF-0.047$ (Fig. 5c^6^). Note that the calibration of MWF was independent of the experimental data in (West et al., 2018b) but based on literature values from an independent experiment. Therefore, $f_{\text{M}\text{W}}$ was used in the following simulations instead of MWF. On the other hand, the calibrated BPF was estimated using the experimental data in (West et al., 2018b). Also note that the $MTV_{\text{M}\text{W}\text{I}}$ requires an intrinsic calibration to normalize the water content (see Section 2.2.1).

In this simulation experiment, we compared $g_{\text{G}\text{T}}$ with the non-calibrated g-ratio values ($g_{\text{M}\text{R}\text{I}}^{\text{n}\text{o}\text{n}\text{e}}$) and with the calibrated g-ratio values ($g_{\text{M}\text{R}\text{I}}^{\text{S}\text{P}\text{C}}$) using either the g-ratio (Fig. 6b,e) or MVF (Fig. 6c,f) single-point calibration (SPC) methods (depicted as scatter in Fig. 6a–c and Bland-Altman plots in Fig. 6d–f). The SPC-reference values were based on the average in control mice (black symbols in Fig. S1: $MVF_{\text{R}\text{E}\text{F}}\approx 0.175$ and $g_{REF}\approx 0.797$). The index MRI described the myelin biomarker that was used to generate the g-ratio: *i.e*. $g_{BPF}$ used BPF (blue crosses), $g_{f_{\text{M}\text{W}}}$ used$\;f_{\text{M}\text{W}}$ (green crosses), and $g_{MTV_{\text{M}\text{W}\text{I}}}$ used ${MTV}_{\text{M}\text{W}\text{I}}$ (black crosses). When calculating the g-ratios, an upper and lower limit was applied meaning that if $g_{\text{M}\text{R}\text{I}}^{2}>1$, the g-ratio value was set to one (because $\left(1-\frac{MVF}{FVF}\right)\leq 1$) and if $g_{\text{M}\text{R}\text{I}}^{2}<0$, the g-ratio was set to zero (because $\left(1-\frac{MVF}{FVF}\right)\geq 0$). In the results, we report the bias and error of the Bland-Altman analyses relative to the dynamic range of simulated ground truth g-ratios: $dyn_{\text{G}\text{T}}=\text{max}\left(g_{\text{G}\text{T}}\right)-\text{min}\left(g_{\text{G}\text{T}}\right)=0.124.$

### Results

The results are summarized in Fig. 6 and Table A1 (in Appendix A). Without calibration, the amplitude of the bias was smallest for the BPF-based g-ratio (23.0 %) and largest for the MTV-based g-ratio (−82.7 %). The error was smallest for the MWF-based g-ratio (13.3 %), moderately larger for the MTV-based g-ratio (20.0 %) and largest for the BPF-based g-ratio (55.1 %). Regardless of single point calibration method (*i.e*. MVF or g-ratio reference) the calibration reduced the bias for the MWF-based g-ratio (≈ −10 %) and the MTV-based g-ratio (≈−22 %) but increased it for the BPF-based g-ratio (≈ 30 %). Importantly, the calibration moderately increased the error for MWF-based g-ratio (≈ 19 %) and substantially for the MTV-based g-ratio (≈ 94 %) but had almost no effect on the BPF-based g-ratio (≈ 56 %). Independent of the calibration, the BPF-based g-ratio showed negative correlation to the ground truth g-ratio, which is caused by the relatively weak dependence of BPF on myelin (Fig. 5a). Note that the abrupt change in the slopes of the MTV-based g-ratio values in Fig. 6 (black crosses) were due to reaching the upper limit for the MR g-ratio (*i.e*. the calibration led to a ceiling effect for this biomarker).

### The relation between MVF and AVF from histology

In this simulation, we used electron microscopy (EM) data in the corpus callosum of mice with hypo- and hyper-myelination as well as in control mice as previously published in (Kelm et al., 2016; West et al., 2018a, 2018b) to establish a heuristic relation between the AVF and the MVF. The functional dependence between AVF and MVF (Eq. (S1) in Fig. S1a) recapitulates the relationship between number of myelin lamellae and axon diameter reported in (Berthold et al., 1983). It is important to note that the heuristic relation in Fig. S1a might hold only for the investigated model mice here and is not necessarily transferable to other scenarios (e.g. variation in healthy white matter of human brains).

## Appendix B Limitations and abbreviations

Table A3

## Footnotes

1 Note that these parameters are for *in vivo* imaging and will be different for ex vivo MRI. For example, in the study by (West et al., 2018a) the low and higher diffusion weighting were at *b*∼3$\frac{ms}{\mu m^{2}}$ and $b∼6\frac{ms}{\mu m^{2}}$, respectively.

2 The aphorism is generally attributed to the statistician George Box, although the underlying concept predates Box's writings (https://en.wikipedia.org/wiki/All_models_are_wrong).

3 This assumption is probably wrong – see model validity in Section 3.1.

4 Note that MTsat cannot be used without calibration, since it is not scaled between 0 and 1. As a consequence we used single-point calibration for both MTsat-based g-ratios in Fig. 7b.
