# Supplementary material for: Towards in vivo g-ratio mapping using MRI: Unifying myelin and diffusion imaging
Source: J Neurosci Methods. 2021 Jan 15;348:108990. doi: 10.1016/j.jneumeth.2020.108990 (PMC7840525; doi:10.1016/j.jneumeth.2020.108990)
Supplement: Supplementary file 1 [file mmc1.pdf]

Figure S1:

(a) Fitting (line) West et al. 2018 data using the heuristic Eq. (S1)

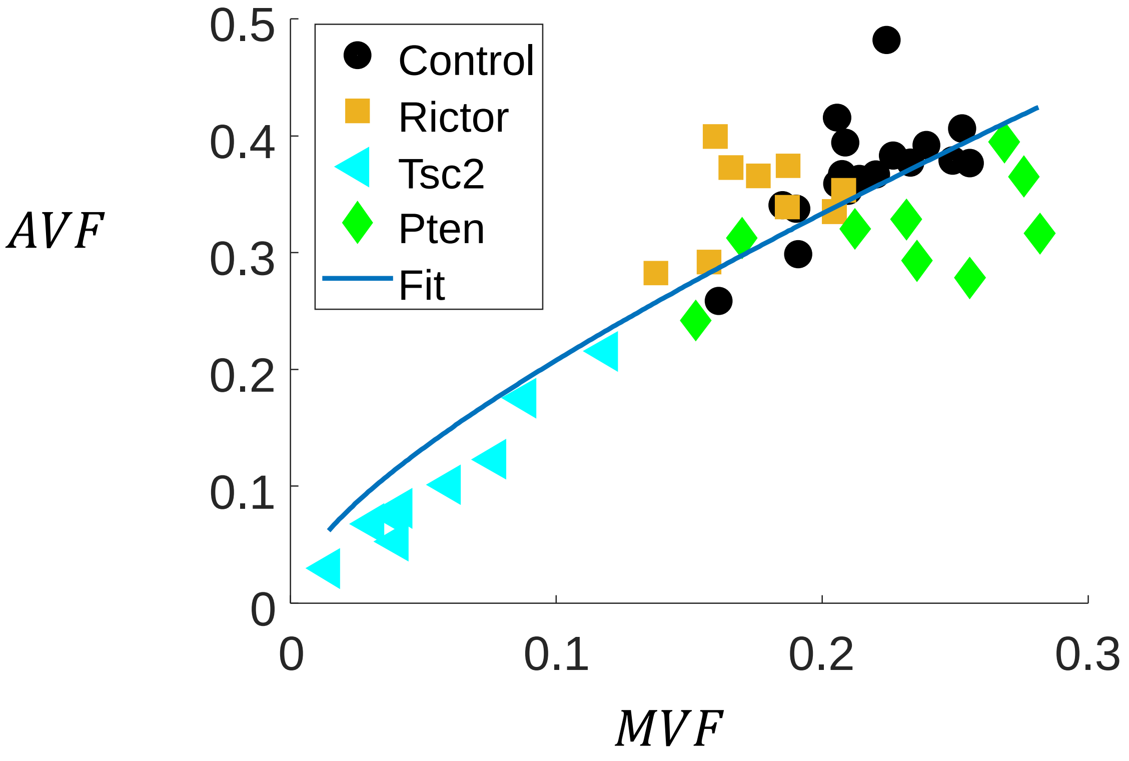

(b) G-ratio calculated from fitted data (using (S2) $g_{GT} = \sqrt{1 - \frac{MVF_{GT}}{MVF_{GT}+AVF_{GT}}}$)

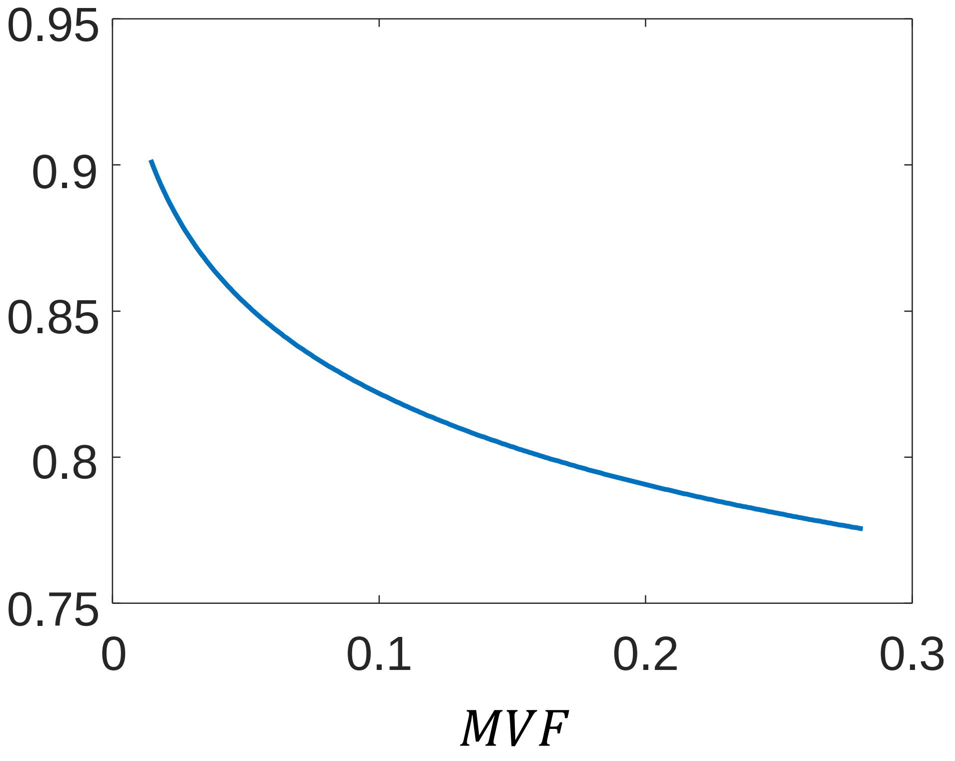

Heuristic equation: $(S1)\ AVF_{GT} = 0.002 + 0.741\ MVF_{GT} + 0.434\ ln(MVF_{GT})$
